# Clinical impact, costs, and cost-effectiveness of hospital-based strategies for addressing the US opioid epidemic: a modelling study

**DOI:** 10.1016/S2468-2667(21)00248-6

**Published:** 2021-11-30

**Authors:** Joshua A Barocas, Alexandra Savinkina, Joella Adams, Raagini Jawa, Zoe M Weinstein, Jeffrey H Samet, Benjamin P Linas

**Affiliations:** Sections of General Internal Medicine and Infectious Diseases, University of Colorado Anschutz Medical Campus, Aurora, CO, USA (J A Barocas MD);Yale School of Public Health, New Haven, CT, USA (A Savinkina MSPH); RTI, Durham, NC, USA (J Adams PhD); Section of Infectious Diseases, Boston Medical Center, Boston, MA, USA (R Jawa MD, Prof B P Linas MD); Boston University School of Medicine, Boston, MA, USA (R Jawa, Prof J H Samet MD, Prof B P Linas); Clinical Addiction Research and Education Unit, Section of General Internal Medicine, Boston Medical Center, Boston University School of Medicine, Boston, MA, USA (Z M Weinstein MD, Prof J H Samet)

## Abstract

**Background:**

The syndemic of injection drug use and serious injection-related infections is leading to increasing mortality in the USA. Although outpatient treatment with medications for opioid use disorder reduces overdose risk and recurrent infections, hospitalisation remains common. We evaluated the clinical impact, costs, and cost-effectiveness of hospital-based strategies to address the US opioid epidemic.

**Methods:**

We developed a microsimulation model to compare the cost-effectiveness of: standard hospital care—detoxification for opioids, no addiction consult service (status quo); expanded inpatient prescribing of medications for opioid use disorder, including bridge prescriptions (ie, medication until they can see an outpatient provider) when possible (medications for opioid use disorder with bridge); implementation of addiction consult services within the hospital (addiction consult services alone); and a combined medication for opioid use disorder with addiction consult services strategy (combined). We used clinical trials and observational cohorts to inform model inputs. Outcomes were life-years, discounted costs, incremental cost-effectiveness ratios, hospitalisations, and deaths. We did deterministic sensitivity analyses on key model inputs related to costs and sequelae of drug use and probabilistic sensitivity analysis to further address uncertainty.

**Findings:**

Among people who inject opioids in the USA, we estimated that expanding medications for opioid use disorder with bridge prescriptions would reduce hospitalisations and overdose deaths by 3·2% and 3·6%, respectively, and the combination of expanded medications with opioid use disorder along with addiction consult sevices would reduce hospitalisations and overdoses by 5·2% and 6·6%, respectively, compared with the status quo. Mean lifetime costs ranged from US$731 400 (95% credible interval 447 911–859 189 for the medications for opioid use disorder strategy) to $741 200 (470 930–868 551 for the combined strategy) per person. Assuming a willingness-to-pay threshold of $100 000 per life-year gained, medications for opioid use disorder with bridge and combined strategies were cost-effective ($7600 and $14 300, respectively). A scenario that assumed ideal access to harm reduction services came to the same conclusions as the base case and our results were robust in deterministic and probabilistic sensitivity analyses.

**Interpretation:**

The combined interventions of expanding hospital-based prescribing of medications for opioid use disorder and implementing addiction consult services could improve life expectancy, be cost-effective, and could be the basis for a comprehensive hospital-based strategy for addressing the opioid epidemic in the USA and countries with similar opioid epidemics.

**Funding:**

National Institute on Drug Abuse and National Institute of Allergy and Infectious Diseases.

## Introduction

The syndemic of injection drug use and serious injection-related infections in the USA is leading to substantial morbidity and mortality. Over time, the opioids driving the crisis have shifted from prescription opioids to heroin and fentanyl, which have shorter half-lives and are predominately injected.^1^ Consequently, complications of injection drug use have risen.^2^ In the past decade, heroin-related deaths have quadrupled^3^ and, in 2019, fentanyl was involved in 50% of the 70 000 overdose deaths.^4^ In addition to overdose, injection drug use places people at increased risk of serious injection-related infections. Endocarditis and skin and soft tissue infections are increasingly common as complications of injection drug use^5^ and incidence of these infections has recently increased markedly.^6^ Unsurprisingly, the costs of the overdose epidemic have also dramatically increased.^7^

Evidence suggests that treatment with medications for opioid use disorder in an outpatient setting greatly reduces overdose and serious injection-related infection risk among people with opioid use disorder, yet, outpatient medications for opioid use disorder are underused.^8^ As such, hospitalisations have become a pragmatic opportunity to both treat opioid use disorder and prevent the associated medical complications, particularly among people who have already experienced overdose or serious injection-related infection.^9,10^ Efforts are ongoing to expand in-hospital medications for opioid use disorder treatment in many ways. One such effort is by providing buprenorphine education for inpatient providers,^11^ which is intended to develop provider competency in buprenorphine prescribing, thereby enabling patients to initiate buprenorphine during hospitalisation and ideally leave with a bridge prescription (ie, medication until they can see an outpatient provider). Buprenorphine education does not equal prescribing—only a fraction of providers actually prescribe buprenorphine—but does help providers gain comfort in managing opioid use disorder.^12,13^

Some hospitals are expanding access to medications for opioid use disorder by implementing addiction consult services.^14^ In addition to access to medications for opioid use disorder, addiction consult services provide counselling, medication dose titrations to long-term (versus acute) regimens, and referrals with linkage to outpatient care.^15^ Many addiction consult services have restricted capacity to care for the growing number of hospitalised patients with substance use disorders.^16^ In addition to providing their own direct care, addiction consult services can support non-addiction providers in managing challenging cases and promote institutional culture change. Therefore, a combination of expanded buprenorphine prescriber workforce and addiction consult services might be a clinically advantageous—albeit costly—model of care, where clinicians can manage more straightforward opioid use disorder cases on their own or with minimal support from addiction consult services, and consult services can manage more complex opioid use disorder cases. Overall, there are disparate approaches by hospitals to providing addiction treatment to hospitalised patients. Some hospitals will encourage inpatient providers to be prescribers without investing in additional infrastructure, whereas some will invest in addiction consult services but without additional support, leaving addiction consult service providers unable to keep pace with increasing volume. Thus, hospitals need evidence-based guidance about the clinical effectiveness and cost-effectiveness of programme strategies that they might consider implementing to address the opioid epidemic. We evaluated the long-term clinical impact, costs, and cost-effectiveness of evidence-based hospital interventions to address the opioid epidemic.

## Methods

### Analytical overview

We used the reducing infections related to drug use cost-effectiveness (REDUCE) model, a validated Monte Carlo microsimulation model that simulates the natural history of injection opioid use,^17^ to compare the cost-effectiveness of four approaches to managing opioid use disorder among hospitalised people, as follows: standard hospital care—detoxification from opioids, no addiction consult service (status quo); expanded inpatient medication for opioid use disorder prescribing with bridge prescriptions (medication for opioid use disorder with bridge); implementation of addiction consult services within the hospital (addiction consult services alone); and combined medication for opioid use disorder with addiction consult services strategy (combined). The model was validated using a three-step approach. First, extensive face validity was sought by various experts in addiction medicine and infectious diseases, including co-authors (JAB, AS, ZMW, JHS, and BPL), to determine that the structure of the model was clinically sound. Next, we internally validated the model by comparing model predicted outcomes, such as average age and proportion of female participants, with the data used to estimate the parameters. Lastly, we assessed external validity using outcomes observed elsewhere (appendix pp 11–13).

In all strategies, hospitalised patients might receive short-term in-hospital medications for opioid use disorder (either methadone or buprenorphine) via inpatient providers while in the hospital, since all providers are covered under a hospital’s Drug Enforcement Administration licence.^18^ In the status quo strategy, patients received medications for opioid use disorder only while inpatients, to address opioid withdrawal symptoms while in the hospital. The other strategies allowed for treatment with medications for opioid use disorder with varying outpatient linkage and intensity of inpatient services.

In the medication for opioid use disorder with bridge strategy, patients might receive inpatient medications for opioid use disorder (buprenorphine or methadone) for treatment and a bridge prescription (if on buprenorphine) upon discharge from a provider who is willing and able to prescribe.^19,20^ However, this strategy lacks a formal linkage mechanism to an outpatient medications for opioid use disorder prescriber.

In the addiction consult services alone strategy, patients might receive inpatient medications for opioid use disorder through an addiction consult in the hospital, which is accompanied by addiction counselling, and enhanced outpatient linkage to an addiction provider who prescribes medications for opioid use disorder. This strategy is informed by existing addiction consult service models in the USA.^15,16,21,22^

In the combined strategy, hospitalised patients might receive medications for opioid use disorder either via an inpatient non-addiction medicine provider or addiction consult service along with benefiting from enhanced linkage to outpatient medication for opioid use disorder care. In this regard, the combined strategy models a synergistic approach to care with increased capacity between addiction medicine and other hospital-based providers (table 1; appendix p 33).

We developed a transition state model to estimate transition probabilities between injection drug use states, stratified by whether someone was on or off medication for opioid use disorder treatment. We used data from the AIDS Linked to the IntraVenous Experience cohort from 1988–2017 and estimated the probability for individuals moving between the aforementioned drug use states. We included only those individuals who reported injection opioid use and months that individuals were on treatment (methadone or buprenorphine) for the on treatment transition probabilities and those timepoints that an individual was off treatment for the off treatment probabilities. Therefore, being on medications for opioid use disorder increased the probability that an individual would transition from a high frequency to lower frequency drug use state (appendix p 8).

The simulated population is a cohort of people who inject illicit short-acting opioids, such as fentanyl and heroin. We used the simulation to estimate outcomes for the cohort including: mortality and hospitalisations attributable to overdose, endocarditis, and skin and soft tissue infections over the lifetime, life expectancy, costs (2020 US$), and incremental cost-effectiveness ratios. We projected lifetime medical costs (including hospital and all outpatient medical care) assuming a payer system perspective and applied a 3% discount rate to both costs and life-years.^23,24^

We used standard methods to calculate the incremental cost-effectiveness ratios of each treatment strategy as the additional cost per person divided by the life-years gained compared with the next least expensive strategy.^23^ We interpreted incremental cost-effectiveness ratios using a willingness-to-pay threshold of $100 000 per life-year gained.^23^

Hospitals do not exist in a vacuum and it is important to understand the value of hospital-based services in different community contexts. Given the varying availability of harm reduction services (eg, syringe service programmes) across the USA,^25^ for the base case we assumed that individuals who inject opioids engage in some routine needle or injection equipment sharing and unsterile injection technique (eg, do not always clean their skin), behaviours that can impact the likelihood of injection-related infections. To improve the generalisability of the model, we completed an alternate scenario analysis that assumed an ideal harm reduction scenario—that people do not share or reuse injection equipment and consistently use sterile injection technique. We also did deterministic and probabilistic sensitivity analyses.

The Consolidated Health Economic Evaluation Reporting Standards guided writing of this manuscript (appendix pp 17–18). The project was reviewed by the Boston University Medical Campus Institutional Review Board and was determined to be non-human subject research, thus individual patient consent was not required.

### REDUCE model structure and inputs

The model is a closed cohort microsimulation of the natural history of injection opioid use, including complications such as overdose, endocarditis, and skin and soft tissue infections (henceforth referred to as sequelae), treatment, and changes in injection behaviours. The model uses a weekly time step (ie, an individual moves through the whole model and then it advances by 1 week) and tracks all individuals from model initiation until death. We simulated the lifetime course of a hypothetical cohort with the demographics of people who inject opioids in the USA, assuming the status quo, which is imperfect access to and availability of harm reduction services, including sterile needles, syringes, and injection equipment. The model includes several modules as described below, in the appendix (pp 2–10), and in a previous publication.^17^

We simulated cohorts stratified by sex, age, and injection behaviour profile. We considered high frequency injection to be at least one injection per day, low frequency injection to be less than one injection per day, and no current opioid use to be no injection use in the previous 12 months.^26^

The model focuses on people who inject opioids. Only those individuals who are in an active injection drug use state experience the sequelae of drug use, with high frequency use carrying a higher risk than low frequency use. We derived overdose and infection risk probabilities by age, sex, and injection behaviour profile.

Individuals who develop sequelae have a probability of being hospitalised and treated for their condition. The key feature of this module is that individuals might encounter various in-hospital services, including inpatient medication for opioid use treatment, consultation with an addiction consult service, or both, depending on the strategy (appendix p 4). Individuals have a probability of being offered and of accepting those services during their hospitalisation. Both initiation of medication for opioid use disorder in the hospital and linkage to ongoing medication for opioid use disorder outpatient receipt affect an individual’s probability of transitioning between injection frequency states, which, in turn, changes an individual’s probability of future sequelae and death. We modelled only buprenorphine and methadone as medications for opioid use disorder and not naltrexone, since the latter might be less effective at preventing overdose and could be challenging to administer to hospitalised patients, especially if they have an ongoing need for opioid medication for pain.^27,28^

Individuals encounter probabilities of linking to outpatient addiction care where they might receive or continue on medication for opioid use disorder treatment. This linkage can happen after a hospitalisation or via a background mechanism. The background mechanism simulates individuals seeking medication for opioid use disorder treatment in the community without first being hospitalised.

Individuals face a risk of death from overdose, endocarditis, and skin and soft tissue infection, as well as from age-related and sex-related causes (ie, background mortality). We derived the probability of a fatal overdose in a given week as the product of the probability of having an overdose based on an individual’s age, sex, injection profile, and the probability of death, conditional upon having an opioid overdose. An additional in-hospital mortality risk is applied during the time an individual is actively being treated in the hospital, which is no longer applied after discharge. If individuals leave the hospital before completion of therapy (ie, against medical advice), they are considered untreated. We apply probabilities of non-opioid-related death from other causes by sex, age, and injection behaviour profile.

Individuals accrue weekly costs related to opioid use, hospital services, and outpatient services. Opioid use disorder care costs vary by injection behaviour profile, with additional costs assigned to those with higher frequency use. There are initial and weekly costs of treatment for opioid use disorder and age-stratified and sex-stratified costs of health-care services that are not attributable to opioid use, endocarditis, skin and soft tissue infection, or overdose.

### Model data

The appendix (pp 11–13) outlines key input parameters for the base case. Population characteristics were derived from the US Census and a combination of published studies.^29–33^

We estimated the rates of fatal and non-fatal overdose from state-level data.^34–36^ We used published literature to estimate the rates of serious injection-related infection and the proportion of infections that are endocarditis and skin and soft tissue infections.^37–40^ The base case scenario assumes imperfect harm reduction services so that individuals are commonly sharing injection equipment and using unsterile injection technique given the lack of available resources, thus increasing their risk of infection. We used published literature to characterise the risk of sequelae.

We used published literature and expert opinion (co-authors JAB, RJ, ZMW, JHS, and BPL) to estimate the rates of hospitalisation for each sequela and to estimate the probability of hospital-initiated medication for opioid use disorder treatment and the estimated effect on injection frequency. Additionally, we used unpublished data from the Boston Medical Center addiction consult services to estimate the probability of receiving an addiction consultation for opioid use disorder among hospitals with an addiction consult service.

We derived the probabilities of linkage to addiction care after hospital discharge and via background mechanism from a combination of cohort studies and clinical trial results.^21,41^

We used overdose-deleted US age-adjusted and sex-adjusted mortality from the National Vital Statistics System^42^ to derive background mortality. Aside from overdose, endocarditis, and skin and soft tissue infection, people who inject opioids might encounter additional drug-related mortality risks (eg, other infections or violence). We accounted for these other mortality risks by multiplying the background mortality by 1·2, which was derived from Chang and colleagues.^43^ To derive the weekly endocarditis mortality, we combined weekly background mortality with weekly mortality risks for people with active injection use (stratified by injection behaviour profile) from overdose,^43^ untreated and treated endocarditis and skin and soft tissue infection,^44,45^ and hospitalisation.^44,46–48^

We assessed costs from the payer perspective in 2020 US$. We derived costs from the 2020 laboratory and physician fee schedules from the Centers for Medicare and Medicaid Services for reimbursement, the Medical Expenditure Panel Survey,^49^ and clinical trials and cohort studies. Costs of drug use were derived from the Clinical Trials Networks Study 0051.^50^

To understand the effectiveness of these interventions under ideal harm reduction circumstances, we did an alternate scenario analysis that assumed no injection equipment sharing or reusing and consistent sterile injection technique.

We did one-way sensitivity analyses to determine the effect of varying model parameters and critical model assumptions. We did probabilistic sensitivity analyses to evaluate the robustness of the results to parameter uncertainty. Distributions were developed for model parameters, and we did 1000 simulations of 5 million people over 100 years while sampling the majority of parameters simultaneously.

### Role of the funding source

The funders of the study had no role in study design, data collection, data analysis, data interpretation, or writing of the report.

## Results

Life expectancy assuming the status quo was 27·19 life-years at an undiscounted lifetime medical cost of US$724 600 per person. Compared with the status quo, medications for opioid use disorder with bridge extended life expectancy by 0·50 years, addiction consult services alone extended life expectancy by 0·94 years, and the combined strategy extended life expectancy by 1·01 years. The status quo resulted in 25 270 hospitalisations per 10 000 individuals over the lifetime, whereas competing strategies resulted in fewer hospitalisations (808 fewer hospitalisations for medications for opioid use disorder with bridge, 1187 fewer hospitalisations for addiction consult services alone, and 1326 fewer hospitalisations for the combined strategy). All scenarios decreased mortality from overdose and serious injection-related infections, with the greatest decrease with the combined strategy (412 fewer deaths per 10 000 individuals over the lifetime; table 2; overdose and serious injection-related infection data not shown).

Compared with the status quo, the average undiscounted lifetime medical cost per person was greater for each of the strategies ($731 400 for medications for opioid use disorder with bridge, $740 500 for addiction consult services alone, and $741 200 for the combined strategy). The combined hospitalisation and outpatient costs decreased with each strategy compared with the status quo, with the largest percent decrease in the combined strategy (−2·3%).

Compared with the status quo, medications for opioid use disorder with bridge had an incremental cost-effectiveness ratio of $7600 per life-year and the combined strategy had an incremental cost-effectiveness ratio of $14 300 per life-year; addiction consult services alone were dominated (provided fewer benefits for money spent—in this case life-years; table 2).

We found that results were not qualitatively different in the alternative scenario of ideal harm reduction; however, the relative improvements in outcomes provided by medications for opioid use disorder with bridge, addiction consult services alone, and medications for opioid use disorder and addiction consult services combined were smaller than in the base case and incremental cost-effectiveness ratios were higher. The status quo resulted in 32·98 life-years at an undiscounted lifetime medical cost of $754 400 per person. Life expectancy was extended by each strategy (by 0·013 years with medications for opioid use disorder with bridge, by 0·032 years with addiction consult services alone, and by 0·038 years with the combined strategy). The status quo resulted in 2598 hospitalisations per 10 000 individuals whereas competing strategies resulted in fewer hospitalisations (13 fewer for medications for opioid use disorder with bridge, 19 fewer for addiction consult services alone, and 21 fewer for the combined strategy). All scenarios decreased mortality from overdose and serious injection-related infections, with the greatest decrease for the combined strategy (16 fewer deaths per 10 000 people). Compared with the status quo, medications for opioid use disorder with bridge had an incremental cost-effectiveness ratio of $10 100 per life-year and the combined strategy had an incremental cost-effectiveness ratio of $20 100 per life-year; addiction consult services alone were dominated (appendix pp 21–22).

**Figure 1:**
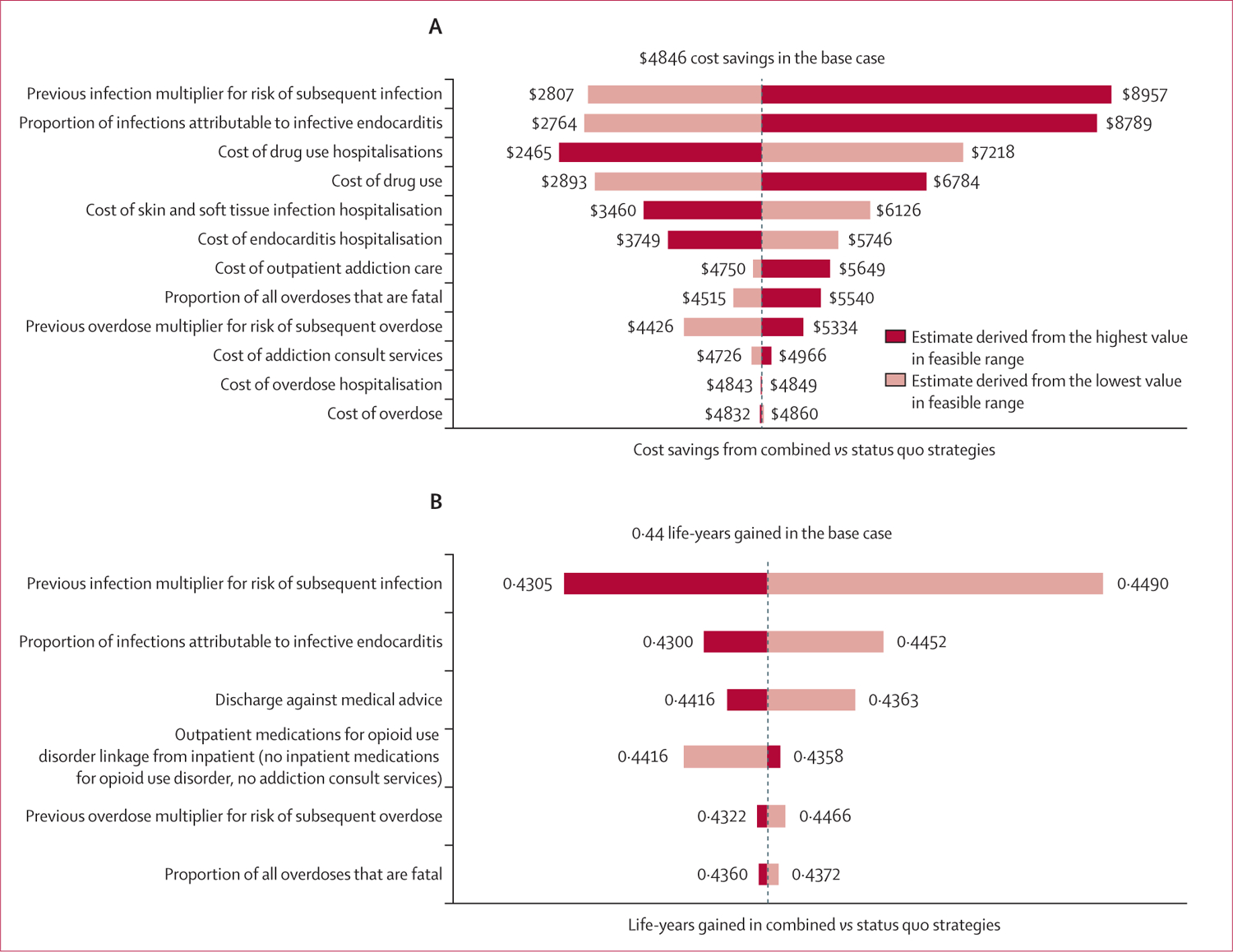
Tornado plots of cost savings (A) and life-years gained (B) from the combined medications for opioid use disorder and addiction consult services strategy compared with the status quo due to parameter changes in one-way sensitivity analysis

We did one-way deterministic sensitivity analysis on parameters that might have affected life expectancy and costs when comparing the combined strategy with the status quo. We found that a history of previous infections and proportion of infections attributable to endocarditis had the greatest impact on costs and life expectancy, and hospitalisation costs had the greatest impact on overall costs (figure 1). We found that 96·6% of patients would need to accept and initiate medications for opioid use disorder via an addiction consult service to make addiction consult services alone a cost-effective strategy (not dominated).

**Figure 2:**
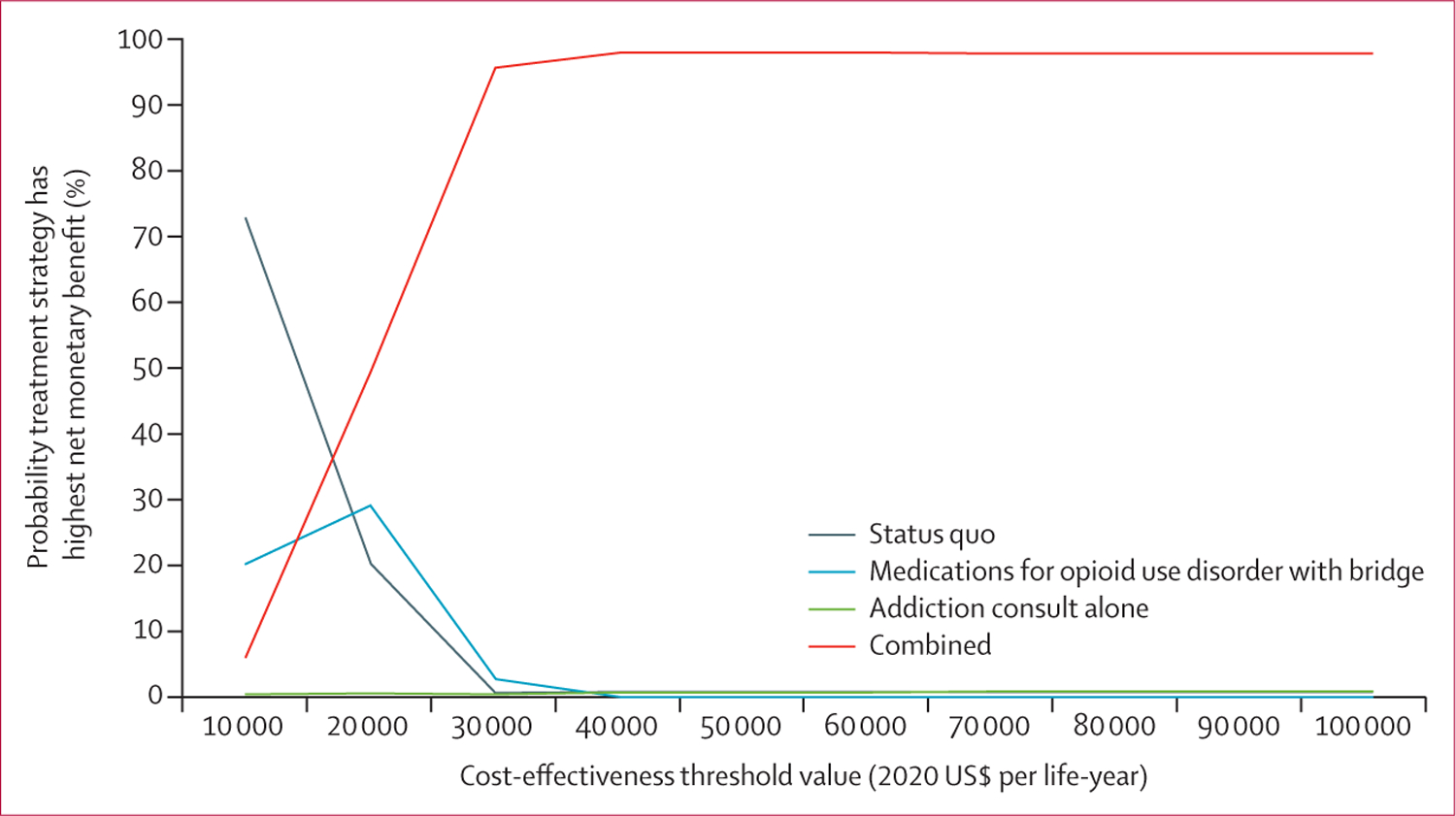
Cost-effectiveness acceptability curves for modelled treatment strategies Cost-effectiveness acceptability curve from probabilistic sensitivity analysis of 990 model runs.

Results in a probabilistic sensitivity analysis comparing the status quo with other strategies were robust in that 98·2% of simulations suggesting that the medications for opioid use disorder with bridge and combined strategies would reduce costs and extend life-years gained (appendix pp 24–27). Results of the probabilistic sensitivity analysis also showed that the combined strategy becomes the preferred strategy at a cost-effectiveness threshold of $30 000 per life-year (figure 2).

## Discussion

In this study, we examined the effect of evidenced-based hospital-based interventions to combat the US opioid epidemic. Our analyses show that in the absence of or in addition to community-based harm reduction services, hospital-based interventions could have a substantial impact on the lives of people who use opioids and on the opioid epidemic.

We showed that expanding medication for opioid use disorder prescribing (via an expanded inpatient non-addiction specialist prescriber workforce) with and without the implementation of an addiction consult service, which also prescribes medications for opioid use disorder and improves linkage to care, are cost-effective strategies. Although investing resources to improve opioid use disorder care in hospitals would be of high value, implementing an addiction consult strategy alone as the sole purveyor of medications for opioid use disorder, without a larger supporting workforce of prescribers, might not be a particularly high value endeavour due to the capacity limitations faced by addiction consult services. In the base case, we assumed that only 25% of people with opioid use disorder would be evaluated by addiction consult services and that 65% of those assessed would initiate medications for opioid use disorder, which might be a conservative assumption. In a threshold analysis, addiction consult services alone only became cost-effective when nearly 97% of patients who were assessed by the services were initiated on a medication for opioid use disorder at that time, which is clinically unrealistic. From clinical experience, addiction consult services are most successful when they arise in a milieu where there are generalist prescribers (who most commonly prescribe buprenorphine treatment) or addiction subspecialty physicians with interest in expanding access to medications for opioid use disorders (including linkage to methadone clinics for those who receive methadone while hospitalised).^21^ Our findings argue further for this combined effort—a strategy with addiction consult services along with an expanded, addiction trained workforce who are interested in prescribing medications for opioid use disorder—as a valuable investment of resources.

Our results, including our probabilistic sensitivity analysis, show that even under ideal community-based harm reduction settings, there are improved clinical outcomes and in-hospital interventions remain cost-effective. Future work should focus on where specific investments should be made within community treatment systems to optimise outcomes.

Our study has limitations. First, this study is a simplification of complex clinical and social processes. Importantly, we used a model of injection drug use with the simplifying assumption that no one returns to oral use after they begin injecting. Additionally, the state transition model was developed from longitudinal self-reported data and such data are not without bias. We explored model assumptions, confounders, and parameter uncertainty with sensitivity analyses, including probabilistic sensitivity analyses. Second, data sources for addiction consult services and other hospital-based interventions were derived from cohort studies and expert opinion; thus, interventions might have differential impacts depending on unique community characteristics. We addressed this issue with an alternate scenario analysis, but it is important to recognise that our estimates should serve as a road map, rather than dogma, to guide hospital decisions. Finally, we modelled only interventions that are used in the US hospital setting. We did not include interventions outside the hospital, such as safe consumption sites, which are not yet sanctioned in the USA.

In conclusion, hospitals have become an important touchpoint for opioid use disorder care, where complications such as overdose and serious injection-related infections can be prevented; thus, hospital administrators and providers must consider strategies to optimally treat this population. Just as it is unacceptable for major hospitals to be without specialist services such as cardiologists, obstetricians, or infectious disease specialists, it should be considered unacceptable to be without addiction treatment services amid an ever-expanding overdose crisis in countries like the USA. As such, hospitals might choose first to expand their workforce for prescribing medications for opioid use disorder, which is a clinically-effective and cost-effective approach compared with the current approach of not addressing opioid use disorder. This initial step should be followed by or done in conjunction with the implementation of addiction consult services. Both strategies together could improve life expectancy, decrease subsequent hospitalisation, be cost-effective, and be the basis for a comprehensive hospital-based strategy for addressing the opioid epidemic.

## Supplementary Material

1

## Figures and Tables

**Table 1:** Description of hospital-based strategies within the simulation model

	Individuals in the model who are eligible for this intervention	Definition	Effect in the model
Addiction consult service	Overdose, skin and soft tissue infections, infective endocarditis, or a combination of these	People who receive an addiction consult benefit from addiction counselling, offer of methadone or buprenorphine, referral and linkage to outpatient addiction treatment including all forms of medication for opioid use disorder and non-medication treatments	Change probability of linkage to outpatient addiction care, change probability of linkage to outpatient medication for opioid use disorder, or change probability of transitioning between injection frequency states
Initiation of medication for opioid use disorder (eg, buprenorphine)	Overdose, skin and soft tissue infections, infective endocarditis, or a combination of these	The medication for opioid use disorder strategy models the impact of non-addiction trained providers prescribing buprenorphine; patients might receive short-term in-hospital methadone (any physician or advance practice provider can prescribe for hospitalised patients), but methadone is not extended beyond hospitalisation	Change probability of linkage to outpatient medication for opioid use disorder, change probability of transitioning between injection frequency states

**Table 2:** Selected cost and clinical outcomes by base case analysis

	Average cost, US$*	Average discounted cost, US$	Incremental average discounted cost, US$	Hospitalisations averted per 10 000 people†	Fatal overdoses averted per 10 000 people‡	Life expectancy, life-years (95% credible intervals)	Discounted life-years (95% credible intervals)	Incremental discounted life expectancy	Incremental cost-effectiveness ratio (US$ per life-year)§
Status quo	724 600 (449 730–845 904)	430 520 (289 000–504 950)	..	..	..	69·19 (61–72)	16·81 (12·88–18·15)	..	..
Medications for opioid use disorder with bridge	731 400 (447 911–859 189)	432 150 (292 319–509 474)	1600	808 (89–784)	37 (16–40)	69·69 (61–73)	17·02 (13·10–18·41)	0·21	7600
Addiction consult services alone	740 500 (468 909–868 206)	434 230 (296 185–513 170)	3100	1187 (0–1354)	59 (14–78)	70·14 (62–73)	17·22 (13·47–18·48)	0·20	Dominated¶
Medications for opioid use disorder plus addiction consult services	741 200 (470 930–868 551)	435 370 (296 848–513 343)	135	1326 (0–1376)	66 (14–87)	70·21 (62–73)	17·25 (13·53–18·48)	0·03	$14 300

* Mean lifetime costs per person with 95% credible intervals.

† Hospitalisations are because of overdose, infective endocarditis, and skin and soft tissue infections, compared with the status quo; total number per 10 000 people with 95% credible intervals.

‡ Fatal overdoses compared with the status quo; total number per 10 000 people with 95% credible intervals.

§ The overall incremental cost-effectiveness ratio was calculated as the difference in the mean discounted costs over the lifetime for the total US population divided by the difference in the discounted quality-adjusted life expectancy for the total US population, all discounted at 3% per year.

¶ Not cost-effective compared with the other strategies.

## Research in context

### Evidence before this study

The US opioid epidemic has evolved from ingestion of prescription opioids to injection of illicitly produced opioids, including heroin and fentanyl. Sequelae of injection opioid use, including overdose and serious injection-related infections, are major drivers of drug use-related morbidity, mortality, and costs. Given their severity, many people are hospitalised for these sequelae. As such, hospitalisation has become an important touchpoint for people who inject drugs to initiate treatment. We searched PubMed for English language articles published up to Aug 23, 2021, with the terms “opioids” AND “injection” AND (“injection-related infections” OR “endocarditis”). This search yielded 23 results. We identified several cohort studies describing the epidemiology of injection drug use and other studies detailing the changing clinical practices addressing substance use disorders within hospital. One simulation modelling study detailed the projected burden of endocarditis, skin and soft tissue infections, and overdose among people who inject opioids in the USA. We found no existing simulation modelling studies estimating the clinical impact, costs, and cost-effectiveness of hospital-based strategies to address the sequelae of opioid use.

### Added value of this study

To our knowledge, this is the first simulation model to evaluate the long-term clinical and cost impacts of hospital-based interventions, such as addiction consult services and expanded inpatient prescribing of medications, for opioid use disorder that might reduce sequelae of injection drug use, such as serious injection-related infections and overdose. Importantly, we also explored the relative value of hospital-based services in different community settings where harm reduction service provision varies.

### Implications of all the available evidence

Our results suggest that a combined approach of expanding access to medications for opioid use disorder in hospital and implementing inpatient addiction consult services could increase life expectancy, decrease hospitalisation, and be cost-effective. This approach could be the basis for a comprehensive hospital-based strategy for addressing the opioid epidemic.
