# Supplementary material for: Clinical impact, costs, and cost-effectiveness of hospital-based strategies for addressing the US opioid epidemic: a modelling study
Source: Lancet Public Health. Author manuscript; Available in PMC 2022 Jan 13. (PMC8756295; doi:10.1016/S2468-2667(21)00248-6)
Supplement: 1 [file NIHMS1768914-supplement-1.pdf]

THE LANCET
Public Health

Supplementary appendix

This appendix formed part of the original submission and has been peer reviewed. We post it as supplied by the authors.

Supplement to: Barocas JA, Savinkina A, Adams J, et al. Clinical impact, costs, and cost-effectiveness of hospital-based strategies for addressing the US opioid epidemic: a modelling study. *Lancet Public Health* 2021; published online Nov 30. http://dx.doi.org/10.1016/S2468-2667(21)00248-6.

Supplemental Appendix

Clinical impact, costs, and cost-effectiveness of hospital-based strategies for addressing the U.S. opioid epidemic

Authors

Joshua A. Barocas, MD, Alexandra Savinkina, MSPH, Joella Adams, PhD, Raagini Jawa, MD, MPH, Zoe M. Weinstein, MD, MS, Jeffrey H. Samet, MD, MA, MPH, Benjamin P. Linas, MD, MPH

Contents

Authors **1**

Contents **1**

Introduction **2**

REDUCE Model **2**

Supplemental Tables and Figures **11**

References **34**

Introduction

The analyses reported in the main manuscript use the Reducing Infections Related to Drug Use Cost Effectiveness (REDUCE) Model of acquisition and treatment for bacterial infections and overdose associated with injection drug use. The REDUCE model tracks several clinical outcomes including number of people with infective endocarditis (IE), skin and soft tissue infections (SSTI), and overdose (OD) (otherwise known as 'sequelae'), number of cases identified, number linked to inpatient and outpatient care, number of people initiating therapy, and number achieving cure from their sequelae of drug use. The model also tracks sequelae-related mortality, quality of life, undiscounted life expectancy, discounted quality-adjusted life expectancy (QALE), discounted lifetime medical costs from the health system perspective, and non-discounted program costs from the payer perspective (for interventions designed to improve follow-up). This technical appendix provides details on key features of the model and modeling approach used for this analysis. We constructed the model and performed analyses using C++ and R (3.2.2). The model is available for review upon discussion with the authors and as resources are available. We did not use every component of the model for the current analysis. In addition, we provide figures and several tables detailing calibration, input parameter values and additional results cited in the manuscript.

REDUCE Model

The REDUCE model is an individual-based, stochastic simulation model of the natural history of injection drug use designed to estimate the outcomes and costs associated with various strategies of prevention, treatment, and improving drug use-related care. The model uses a cycle length of one week. Injection frequency is usually reported as injection frequency in the past month" and while frequency may change daily depending on drug availability, most frequency use is somewhat stable over a one-week period.

Overview. The model is designed as a number of modules through which simulated individuals pass. Briefly, a cohort module helps to "create" the population of interest. Next, individuals created during cohort generation enter the "sequelae of drug use (SDU)" module, which is where they encounter probabilities of fatal or nonfatal overdose, infective endocarditis, or skin and soft tissue infections. From the SDU module, individuals enter back into the simulation or link to the "inpatient" module. In the "inpatient" module, individuals are hospitalized for their SDU. There are a variety of interventions (beyond standard hospital treatment) that individuals may encounter if those services are turned "on" by the user. Following the inpatient module, individuals have a probability of linking to outpatient care in the "outpatient" module. Linkage to outpatient care may vary based on the type of services an individual encountered in the hospital and/or the type of SDU they have (overdose vs infection). They may unlink from the outpatient module or never enter it (based on probabilities). The "behavioral transitions" module is when individuals have the probability of moving between injection frequency drug use states (high frequency, low frequency, or no current drug use), between sterile injection practice states (skin cleaning or no skin cleaning), and sharing/reusing needles. After the "behavioral transitions" module, individuals move to the "mortality, cost, and quality of life" module. At this point, the model begins again in cycle n+1.

Module 1: Cohort initiation. When the model is initiated, a cohort of individuals is generated using 6 parameters:

(1) ever injection drug use status (ever/never)
(2) age (0-99).
(3) sex (M/F)
(4) injection frequency (high/low/no current/never)
(5) reusing/sharing equipment (yes/no/never).
(6) sterile injection practice (cleaning/no cleaning/never)

From these parameters, the cohort developed is a distribution of people who have ever or never injected drugs and those who are ever are stratified by injection frequency and injection practices. The model is structured such that first the user specifies the proportion of the population that has ever injected drugs. Following that, there are two methods by which the model can draw age and sex. The first is by using age/sex tables and the second is by directly specifying age and sex distribution parameters. In the latter method of drawing from age and sex, the user inputs values directly into the deterministic parameter file. These inputs include proportion male, average male age, standard deviation male age, average female age, standard deviation female age, and minimum age

Next, among those who are ever drug users, the probability of injection frequency is drawn from an age/sex stratified table—high, low, and no current injection drug use. For this, all three probabilities of an age/sex group should equal to 1 and the model draws from this set of probabilities. The model does not allow for the added probability to be greater than 1. Finally, all persons who are ever drug users, are assigned an initial status of being a

skin cleaner and a needle sharer which does not depend on age and gender. While these are the initial attributes, all individuals have the possibility of "picking up" additional attributes as they move through the model. All never drug users are assigned "never" injection frequency, skin cleaning and needle sharing status.

Assumptions built into the model for the initial cohort:

1) no one starts on treatment for opioid use disorder
2) no one starts out with a history of overdose
3) no one starts with a history of infection
4) no one begins in care or in the hospital setting.

Module 2. Sequelae of Drug Use. Once the cohort is initialized and each individual has been assigned an initial drug use status, age, sex, and injection frequency and practices, individuals enter the sequelae of drug use (SDU) module. Broadly, the SDU in this model include infective endocarditis (IE), skin and soft tissue infections (SSTIs), and overdose (OD). When they first enter, the model checks their ever/never status. If they are "never," then they return to the simulation. Therefore, only "ever" drug users can progress through this module. The model then checks their injection frequency. If they are "no current," then they return to the simulation. Therefore, only "low frequency" and "high frequency" injectors progress through this module. Additionally, if the individual is currently in inpatient care, they return to the simulation. If a person is currently on antibiotics, they progress through the SDU module but they cannot acquire a new infection (SSTI or IE).

At this point, remaining individuals are subject to probabilities for acquiring an SDU. On the first cycle of this model, no one has a history of SDU, but have the possibility of acquiring one or multiple through their life. History of SDU is tracked as it has implications for future SDU. One assumption of the model is that in each cycle, an individual can have more than one SDU, but only acquires one infection at a time. Additionally, individuals may have concurrent SDU (meaning that they may acquire IE in cycle 1 and then SSTI in cycle 2, if they have not been hospitalized for their existing SDU. Another assumption of the model is that SDUs can only be acquired while not "inpatient" or on antibiotics (next module).

Individuals who are eligible for an SDU, progress through a number of probabilities of acquiring an SDU. All SDUs are stratified by injection frequency (high and low) and the infectious SDU are also stratified by injection practices (skin cleaning, needle sharing). SDU probabilities are not stratified by age and sex. The model is structured such that an individual first encounters a combined probability of overdose (fatal + nonfatal), stratified by injection frequency. If an individual has a current infection their overdose rate is multiplied by the current infection multiplier. A proportion of overdoses are fatal and a proportion are nonfatal. If a person draws an overdose, the model checks whether they are within their OEND effective cycles and dependent on that, fatal overdose is drawn. One aspect of the model is that at this point, if a person draws a fatal overdose then they are flagged as "dead, fatal overdose." They continue to proceed through the rest of the modules but cannot acquire any further attributes (e.g., they cannot get another infection, be hospitalized, start MOUDs, change their behaviors). These individuals, however, accrue the full costs of the cycle (based on background costs, costs of fatal overdose, and costs of any other SDUs that are untreated) and utilities (based on age, sex, and other current health states at the end of the cycle). For those that have a nonfatal overdose or do not have an overdose, they then face a combined probability of infectious SDU (IE + SSTI), stratified by injection frequency, skin cleaning and needle sharing attributes. A proportion of that probability is IE and the other is SSTI. The model is structured to account for a history of SDU (treated in hospital, resolved because it was a nonfatal OD) and for existing SDUs. An existing SDU is anything that an individual has during the current cycle. From a clinical perspective, this represents an "untreated" infection (e.g., someone has not gone to the hospital for their endocarditis or someone is currently on outpatient antibiotics but not cured) or a current nonfatal overdose. Once treatment is complete or the SDU resolves (as is the case with nonfatal overdose which resolves in 1 cycle), then the person is flagged with a history of the corresponding SDU. Individuals with a history of endocarditis, for example, do not continue to accrue morbidity-associated costs after the endocarditis is resolved. Put another way, costs are time updated in the model such that costs associated with sequelae only last for the duration of the sequelae.

An existing SDU causes a change in the likelihood of another SDU. In the model, there is a single multiplier for one or more existing SDUs that is applied to both the probability of OD and the probability of infectious SDU. This multiplier exists until the individual is treated for the SDU. For those who have a nonfatal overdose, the existing SDU multiplier will be applied to the probability of infection in the same cycle only since an existing nonfatal OD (that does not link to inpatient), only lasts one cycle. Additionally, a history of SDUs changes the probability of

future SDUs. Multipliers are only applied to the SDU for which there is a history (e.g., OD history changes the probability of recurrent OD; any infection history changes the probability of future infection [any infection, not just the one that occurred]). For OD, there are 4 multipliers (e.g., 1 past nonfatal OD, 2-3 past nonfatal OD, 4-7 past nonfatal OD, and 8+ past nonfatal ODs). For history of treated infections, there is only one multiplier (1+ past treated infections). For instance, in cycle 1, an individual gets IE but does not go to the hospital/receive treatment and does not die in cycle 1. By cycle 2, having IE makes that individual have a greater probability of OD or SSTI. For this model, individuals will not be able to acquire the same SDU in that next cycle. From the previous example, the individual with IE will only be able to acquire OD or SSTI, not IE in cycle 2. While that infection remains untreated, there is an effect on getting another infection/OD. Once that infection is treated, then there is a separate effect of this infection on future infections. Therefore, this module has two multipliers: 1) one that can change the probability of an additional SDU if current SDU is untreated, and 2) one that can change the probability of a recurrent SDU (in the future) if the current SDU is fully treated and they survive it.

If an individual does not acquire an SDU in the current cycle and does not have an untreated SDU from a past cycle, they return to the simulation. If they acquire one or more SDUs, or have an untreated SDU from a past cycle, then individuals draw linkage probability to inpatient from the SDU. Linkage to inpatient depends on the linkage probability of their SDU; if an individual has more than one SDU, their linkage probability is the highest of the linkage probabilities for the SDUs they have. There remains the possibility that an individual does not link to inpatient. In the case of nonfatal OD, it implies that the OD was not severe enough to require hospitalization (or was treated in the field). In the subsequent cycle, there should not be a flag for untreated overdose. All nonfatal overdoses are, by definition, treated so the "existing" state can only last for the cycle in which the non-fatal overdose occurs. In the case of endocarditis, the untreated flag should remain on until the person either dies or links to inpatient care and gets cured. This is because endocarditis is generally uniformly fatal if untreated. In the case of SSTI, some SSTIs can spontaneously clear (e.g., consider a pimple or slight redness around a cut). In the model, we are assuming that SSTIs being modeled are serious infections that would require hospitalization or, otherwise, ultimately lead to death. Therefore, similar to IE, the untreated SSTI flag remains on until either 1) the person dies, or 2) the person is linked to inpatient and cured, whichever occurs first. Individuals who go to the hospital will be classified as "inpatient" starting in the same cycle and will have an "in-hospital mortality." Once they leave the hospital, they are considered as having a history of infection. If an individual does not link to inpatient, they are classified as having "existing" SDU and have different risks of death (untreated mortality probabilities for each SDU). Individuals who come to the SDU module on subsequent cycles with an additional SDU (>1 SDU at a time) will have the probability of hospitalization that is equal to the highest probability of the SDUs.

Attributes that an individual can acquire in this module and are tracked:

1. Current IE
2. Current SSTI
3. Current OD, non-fatal
4. Current OD, fatal
5. History of treated IE
6. History of treated SSTI
7. History of treated OD

Module 3. Inpatient Hospitalization Module. One assumption of the model is that any individual that is either a) current injection drug use or b) has a current, untreated SDU is presumed to have opioid use disorder (OUD). Some sequelae of OUD are infectious and some are non-infectious (e.g., overdose).

Each individual with 1+ SDU has a probability per cycle of presenting to an inpatient setting for their care. When individuals enter the inpatient module, the model checks their current SDU status. If they do not have a current untreated SDU or died of fatal overdose in the previous module, or they are on outpatient antibiotics, then they return to the simulation. Therefore, only those individuals with active SDU can progress through this module.

The path through the inpatient module is conditional on the SDU(s) that an individual has: nonfatal OD, IE, SSTI, or combination. The hospitalization duration for overdose is 1 cycle; the hospitalization duration for SSTI and IE are drawn stochastically from a normal distribution with a user defined mean and standard deviation; the model allows for a maximum hospitalization to be set so that at the end of the max amount of time a person will leave the hospital. Each hospitalization is associated with a cost that is accrued in later module. The key feature of this module is that individuals may encounter a variety of in hospital services. These services are either turned on or off by the user

depending on the analysis. If they are on, then individuals will have a probability of being offered and of accepting those services during their hospitalization. Each service has an effect either within this module or elsewhere in the simulation. Each service is associated with a cost that is applied in a separate module at the end of the simulation. Individuals should be "marked" as using/receiving a service such that the cost can be tabulated in the separate module. Additionally, some of the services have an independent effect on quality of life. Similar to cost, this is applied in a separate module at the end of the simulation. Hospitalization is associated with a decreased QoL so there is a hospitalization QoL weight that can be applied in a separate module at the end of the simulation.

**Hospital-based service**	**SDU for which service applies (eligibility)**	**Effect in the model**	**Independent cost**	**Independent quality of life**
Addiction consult service	OD, SSTI, IE, combination	Change (increase) probability of linkage to outpatient addiction care*, changes probability of linkage to outpatient MOUD. Change probability of transitioning between injection frequency states. See treatment effect description below.	Yes, this recurs on a weekly basis while inpatient	No
Initiation of MOUD (e.g., buprenorphine)	OD, SSTI, IE, combination	change probability of linkage to outpatient MOUD. Change probability of transitioning between injection frequency states. See treatment effect description below.	See treatment effect. Yes, recurs on a weekly basis while inpatient	Yes

*These interventions are applied only in the last cycle of hospitalization and they will have post-treatment effective cycles drawn from a normal distribution.

Each individual has a probability of in-hospital mortality that is discussed in detail in the mortality module section. It is mentioned here to note that it is an attribute that an individual can acquire. During hospitalization, individuals "carry" a flag/marker that designates them as hospitalized. While hospitalized, individuals cannot get a new SDU so they will not enter SDU module. They have an "in hospital" mortality that is conditional on the SDU for which they are hospitalized. For the duration of their hospitalization, their injection frequency is considered to be "no current" regardless of their actual status and they are not exposed to behavior transitions. The exception to this rule is as follows: In the last hospitalization cycle, individuals are exposed to behavior transitions based on their pre-hospitalization status. If they have received any intervention that would affect their behaviors (MOUD, skin cleaning education or clean needle distribution), the intervention effect will be applied to their actual or pre-hospitalization behaviors and post-treatment effective cycles will be drawn. These behavioral changes are assigned in the last inpatient cycle so that they take effect the first cycle out of inpatient. However, cost-life-mortality module still consider them as "no current". When the inpatient hospitalization time has lapsed, then individuals move to the outpatient module. In the outpatient module, they have a probability of then linking to different types of care.

For the current analysis, needed to first derive the following probabilities: 1) addiction consult service, probability of uptake if available, 2) Initiation of MOUD, probability with an addiction consult, and 3) Initiation of MOUD, probability without an addiction consult. For 1): we used personal communications with Zoe Weinstein who leads the BMC addiction consult service and Caroline King who has analyzed unpublished data from the addiction consult service at OHSU to derive the percent of people who get ACS. Dr. Weinstein's initial data estimated that approximately 25.8% of those individuals who have opioid use disorder receive an addiction consult service. This is a conservative estimate of the number of patients with opioid use disorder that may be seen by an addiction consult service, therefore we did a sensitivity analysis that varied this percentage from 4% to 40%. For 2): we used unpublished data from the BMC and OHSU addiction consult services, which demonstrated that approximately 65% of people with an OUD (range 32-97%) who receive an addiction consult get started on methadone or buprenorphine. For 3) We used data from the VA Health System to estimate that 11% (range 5-16%) of people admitted with OUD received MOUDs without ACS. Therefore, for scenario of MOUD without ACS, the overall probability was 11%. For scenario of ACS which can offer MOUD, we multiplied 25.8 by 65% =16.9. This means that approximately 25% of people with OUD get seen by ACS and that 65% of those get started on MOUD by ACS.

For the combined strategy, we added the combined probability of people with OUD getting MOUD without an ACS with the probability of people with OUD getting an MOUD with ACS (11 by 74% + 25.8 by 65%=25%). We then divided all of these probabilities by 3 to obtain weekly probabilities since the average length of stay was 3 weeks.

	ACS Percent	MOUD no ACS	MOUD Percent on ACS
**Lower Range**	0.04	0.05	0.32
**Model Param**	0.258	0.11	0.65
**Upper Range**	0.4	0.16	0.97

In model (accounting for avg LOS)	MOUD no ACS	MOUD on ACS only	MOUD and ACS
**Lower Range**	0.016666667	0.004266667	0.020266667
**Model Param**	0.036666667	0.0559	0.083106667
**Upper Range**	0.053333333	0.129333333	0.161333333

Module 4. Outpatient Care Module. There are two different ways in which an individual can enter the outpatient module. First, an individual can enter via background linkage. This means that those who are not hospitalized but "decide" to seek care can do so by entering this module. Second, an individual can enter via the inpatient module.

For individuals entering from the simulation (background). Each individual encounters the outpatient module. Individuals with a "death" flag from a previous module (fatal overdose) enter the outpatient module and immediately return to the simulation. Individuals who are currently hospitalized immediately return to the simulation. All other "ever" drug user individuals have a probability of linking to outpatient care and progress through the outpatient module, regardless of history of SDU or drug use status. If individuals do not draw "linkage" then they return to the simulation. *For individuals entering from the inpatient module (inpatient linkage).* When the inpatient hospitalization time has lapsed, then individuals encounter a linkage probability to the outpatient module depending on inpatient services they have received.

<u>Outpatient addiction care</u>. Individuals have a probability of linking to outpatient addiction care (either with or without MOUDs). One cannot be simultaneously in outpatient addiction care with MOUDs and without MOUDs (these are separate states). But individuals can be simultaneously in outpatient addiction care (with or without MOUDs) and outpatient antibiotics.

Individuals have a probability of unlinking from outpatient addiction care either with or without MOUDs or transitioning between MOUD states. There is a separate probability of linking to outpatient addiction care (with or without MOUDs) for those coming from the inpatient module and those coming from the simulation (spontaneous linkage/background linkage). There are different linkage probabilities for the following groups:

1. Individuals who have received inpatient addiction care but did not get MOUD
2. Individuals who have received inpatient addiction care and got MOUD
3. Individuals who did not receive inpatient addiction care but got inpatient MOUD
4. Individuals who did not receive any relevant inpatient services or individuals coming from the background (no hospitalization)

If an individual is in outpatient addiction care and acquires an infection (SSTI or IE) they will automatically be linked to inpatient care in the next cycle. In this case, they will unlink from outpatient care and all outpatient related flags/cycles will be cleared.

**Outpatient service**	**Eligibility**	**Effect in the model, while linked**	**Independent cost**	**Independent quality of life**
Outpatient addiction with MOUD	Any ever IDU in the simulation not actively hospitalized	Change (decrease) probability of unclean injection; change (decrease) needle sharing, change (increase) probability of moving to lower frequency state and change (decrease) probability of moving out of no/low frequency state. See treatment effect document.	Yes	Yes
Outpatient addiction without MOUD	Any ever IDU individual in the simulation not actively hospitalized	Change (decrease) probability of unclean injection; change (decrease) needle sharing	Yes	No

Module 5: Behavioral Transitions Module. Following the inpatient and outpatient modules, individuals move to the behavioral transitions module. Individuals may also enter this module "from the simulation." The latter represents the ability of someone to change their behaviors organically (without interventions). This is the module in which they can move between high frequency, low frequency, and no current use states, move from never and ever IDU, move between skin cleaning and not skin cleaning states, and move between sharing needles and not sharing needles states. There is a prior probability of movement between states (status quo) and various "flags" acquired throughout the model progression that impact certain probabilities. These have been outlined in various other module descriptions but are also be outlined below.

Treatment Effects: The primary driver of morbidity and mortality in the module is the injection frequency. High frequency individuals are at higher risk than low frequency injectors of sequelae of drug use (SDUs), which include overdose, skin/soft tissue infections, and endocarditis in this model. All persons who are "ever" injectors have the possibility of moving to a higher or lower injection frequency state (depending on their current state) or staying in their current state per cycle. For instance, a high frequency injector may remain as a high frequency injector or may move to low frequency or no current use states. There are a few ways that the injection frequency can be modified in the model. In brief, however, only hospitalization and MOUDs can change injection frequency in the model.

Mechanisms by which transitions between injection frequency states are changed:

1) Hospitalization.
2) Outpatient MOUD initiation.
3) Inpatient MOUD initiation.
4) Behavioral transitions with MOUD.

	Frequency state					
Treatment status	HF→LF	HF→No	LF→HF	LF→No	No→HF	No→LF
No treatment						

Immediate MOUD effect (0-4 weeks)						
>4-week MOUD effect						

For this analysis, we developed a transition state model to estimate transition probabilities between injection drug use state, stratified by whether someone was on or off treatment. We used data from the ALIVE cohort from 1988-2017 and estimated the probability for individuals moving between the aforementioned drug use states. We included only those months that individuals were on treatment (methadone or buprenorphine) for the "on treatment" transition probabilities and those time points that an individual was off treatment for the "off treatment" probabilities. Being on MOUD, therefore, increased the probability that an individual would transition from a high frequency to lower frequency drug use state. Those values are noted below:

No Treatment (Weekly Probabilities)									
	none_to_low	none_to_high	none_to_none	low_to_none	low_to_high	low_to_low	high_to_none	high_to_low	high_to_high
estimate	0·0032	0·0015	0·9953	0·0089	0·0083	0·9828	0·0042	0·0074	0·9884
lower_ci	0·0030	0. ·0015	0·9955	0·0086	0·0080	0·9834	0·0040	0·0072	0·9888
upper_ci	0·0033	0·0016	0·9951	0·0092	0·0086	0·9822	0·0044	0·0077	0·9880

Combined Treatment (Weekly Probabilities)									
	none_to_low	none_to_high	none_to_none	low_to_none	low_to_high	low_to_low	high_to_none	high_to_low	high_to_high
estimate	0·0107	0·0117	0·9776	0·2367	0·0248	0·7384	0·1617	0·1617	0·6766
lower_ci	0·0013	0·0038	0·9949	0·1606	0·0102	0·8292	0·1534	0·1534	0·6932
upper_ci	0·0177	0·0258	0·9566	0·2454	0·1538	0·6008	0·2281	0·2281	0·5439

Module 6: Mortality Module. The mortality module also includes costs and quality of life adjustments.

Mortality. There are two places in the model that an individual can die: fatal overdoses in the SDU module and in the mortality module. To review, in the SDU module, an individual draws a combined probability of all types overdose which is stratified by injection frequency (high and low frequency). From that combined probability, an individual can draw either a fatal or non-fatal overdose. If an individual draws a fatal overdose, then go through the remainder of the cycle with a "fatal OD" flag up which does not allow them to get any further interventions, collect additional costs, change their behavior status, etc., however, they will accumulate the background cost and utility of that cycle. As such, the background mortality in the mortality module should exclude overdose mortality.

The background mortality risk is an age and sex adjusted mortality probability (excluding fatal overdose). There are a number of occurrences in the model that can impact the weekly risk of mortality. First, individuals who are

hospitalized for an SDU (non-fatal overdose, SSTI, or endocarditis) have an increased risk of death. If the inpatient individual further gets an ID consult, their infection inpatient mortality rate is augmented by an ID consult mortality multiplier (ID consult will not affect overdose mortality). Second, individuals who have an untreated skin and soft tissue infection or untreated infective endocarditis have an increased risk of death. These risks are input as probabilities (and converted to rates by the model) which are then *added* to the background mortality at the end of each cycle. Once a patient is cured of their infection their SDU flags are removed and their mortality goes back to background mortality. The mortality risk only applies for each cycle that they have that risk. For example, a person gets endocarditis and does not present to inpatient care during a cycle. Then they have an "existing endocarditis" flag that the end of the cycle should prompt the rate of death for untreated endocarditis to be added to the background mortality. On cycles 2-5 that same individual, however, is hospitalized and being treated for their endocarditis. For those cycles, they get an "in-hospital for endocarditis" flag such that the in-hospital endocarditis mortality rate is added to their background mortality each cycle. On cycle 6, this person leaves the inpatient setting (completes treatment) so all flags are, therefore, off and at the end of that cycle they get only background mortality. We do not include an additional mortality risk for being an active drug user since most of that risk will be folded into overdose and other SDUs.

<u>Cause of death as an output</u>: In the model, individuals can die of background causes or as a direct result of their injection drug use. Direct causes of injection drug use include:

1. Overdose (combination of fatal overdose/ hospitalized and nonfatal OD that dies in the hospital)
2. endocarditis (combination of hospitalized and non-hospitalized)
3. SSTI (combination of hospitalized and non-hospitalized)

Aside from fatal overdose, all of the other causes of death get added to the background mortality as outlined above. For instance, an individual's weekly probability of death (conditional on not dying of a fatal overdose) may be p_d and they may have endocarditis which increases their risk of death by x. The individual's weekly risk of death is, therefore, *the sum of the rates converted to a probability.* However, as an output, we need to be able to determine the attributable cause of death (this person may have died of endocarditis OR background causes). To do this, we use the sum of the rates as the denominator and the individual mortality risk (rates) as the numerator in drawing the cause of death. Important for consistency, the input parameters are probabilities and therefore all rates are calculated in the model. For instance, background mortality rate is x (prob = p_x), untreated endocarditis is y (prob = p_y), and untreated SSTI is z (prob = p_z). Total rate = x + y + z. This is converted to a probability of death, p_c. If death = yes, then to determine the cause, the model draws from the following probabilities: probability that death was from background = r_x/r_c; probability that death was from endocarditis = r_y/r_c; probability that death was from SSTI = r_z/r_c (Cause of death should be calculated based on rate ratios) The same methodology would hold true if there were two concurrent SDUs. As outputs, we only want to output background deaths, endocarditis deaths, SSTI deaths, and overdose deaths (do not need to stratify by inpatient vs outpatient).

Costs. Costs are accrued for a variety of reasons. At the end of each cycle, costs associated with certain characteristics should be added up to the background costs. A discount rate should be applied at that time.

1. Background costs: age and sex stratified costs of being alive which are the same for never and ever IDUs
2. Injection drug use costs: Ever injection drug users should have costs that are stratified by frequency:
    a. Cost of no current injection drug use
    b. Cost of high frequency injection drug use
    c. Cost of low frequency injection drug use
3. Cost of fatal overdose
4. Cost of non-fatal overdose not hospitalized
5. Cost of untreated endocarditis
6. Cost of untreated skin/soft tissue infection
7. Per cycle costs of hospitalization for endocarditis*
8. Per cycle costs of hospitalization for skin and soft tissue infection*
9. Per cycle costs of hospitalization for overdose*
10. Inpatient services costs
    a. Addiction consult service: recurring weekly cost while inpatient
    b. MOUDs: recurring weekly cost while inpatient
11. Outpatient services costs

a. Outpatient addiction with MOUD: recurring weekly cost while on MOUD and linked to addiction care
b. Outpatient addiction without MOUD: recurring weekly cost while linked to care

*If someone is hospitalized for multiple causes (IE, SSTI, OD) they do not get costs for all 3 as we would be double (or triple) counting. Instead, they get the maximum of the hospitalization costs for what they have (i.e., if the individual was hospitalized for SSTI and IE, they would receive whichever costs are higher, SSTI or IE, but not costs for both).

Health utilities. There are two ways that health states can be calculated: 1) minimum estimator and 2) multiplicative approach. In the minimum estimator approach, all of the health state utilities are looked at by the model individually and the lowest health state utility is "chosen" to be the utility for that particular cycle. In the multiplicative approach, all of the health state utilities that apply to a given individual are multiplied together to come up with a new, unique health state utility. These utilities get applied to the life expectancy to come up with a quality-adjusted lifespan (quality adjusted life years, expectancy, etc). Health states with different utilities in this model:

1. Age and sex stratified utility (ages 0-100, men and women)
2. Utility of being an ever drug user in no current drug use
3. Utility of being an ever drug user in low frequency drug use
4. Utility of being an ever drug user in high frequency drug use
5. Utility of nonfatal overdose, not hospitalized
6. Utility of untreated endocarditis
7. Utility of untreated SSTI
8. Utility of being hospitalized endocarditis
9. Utility of being hospitalized skin infection
10. Utility of being hospitalized OD
11. Utility of being on MOUDs

All costs and life expectancy should have a discount rate applied at the end of the cycle so that we can derive a discounted cost and a discounted quality adjusted life expectancy.

Supplemental Tables and Figures

Supplemental Table 1. Estimates for Key Model Parameters to Characterize Outcomes of People who Inject Drugs over a Lifetime

Parameter	Estimate	Range	Source
**Population**			
Probability of ever drug use (one-time)	Varies by age and sex		[1-5]
Probability of injection drug use frequency (one-time)	Varies by age and sex		[6,7]
**Sequelae of Drug use** (weekly)			
Probability of overdose			
Low frequency drug use	0·0026	0·0026-0·0027	[4,8-12]
High frequency drug use	0·0005	0·0005-0·0006	[13]
Probability of fatal overdose	0·1300	0·1200-0·2400	[12-14]
Combined probability of IE and SSTI, stratified by injection behavior profile	Varies (Supplemental Appendix)		[15-20]
Proportion of infections attributable to IE	0·1300	0·0300-0·4000	[15,16,21,22]
Probability of linking to inpatient care following nonfatal overdose	0·9700		Unpublished data[****]
Probability of linking to inpatient care for IE	0·2000	0·1830-0·2170	[23]
Probability of linking to inpatient care for SSTI	0·0019	0·0008-0·0040	[24]
Previous overdose multiplier for risk of subsequent overdose	1 NFOD: 1·15	0·72-1·82	[25]
	2-3 NFOD: 1·81	1·19-2·27	
	4-7 NFOD: 2·12	1·11-4·04	
	8+ NFOD: 5·24	1·56-17·01	
Previous infection multiplier for risk of subsequent infection	2·8	1·50-5·10	[26]
**Inpatient**			
Duration hospitalization SSTI (weeks), mean	2	1-4	[16]
Duration hospitalization IE (weeks), mean	6	4-8	[16]
Probability of AMA discharge (weekly)	0·0500	0·0300-0·1000	Expert opinion
Addiction consult service, probability of uptake if available (weekly)	0·2580	0·04-0·40	Unpublished BMC ACS data; expert communication[*] (See Supplemental Appendix)
Initiation of MOUD, probability with an addiction consult (weekly)	0·6500	0·32-0·97	Unpublished ALIVE[**] data; [27-29] (See Supplemental Appendix)
Initiation of MOUD, probability without an addiction consult (weekly)	0·1100	0·05-0·16	
**Outpatient** (weekly)			
**Outpatient addiction care linkages**			
Outpatient addiction care (MOUD) from inpatient with ACS and MOUD	0·7000	0·6700-0·7220	Unpublished data[***], [30,31]

Outpatient addiction care (MOUD) from inpatient without ACS, with MOUD	0·5714	0·5404-0·6024	Unpublished data***
Outpatient addiction care (No MOUD) from inpatient addiction care with ACS, without MOUD	0·4529	0·4415-0·4643	Unpublished data***
Outpatient addiction care from inpatient care (No MOUD), without ACS	0·0500	0·0490-0·0501	[32,33]
**Outpatient MOUD initiation**			
Outpatient addiction care from inpatient with ACS	0·5069	0·4649-0·5489	Unpublished data***
Outpatient addiction care without ACS	0·1620	0·1439-0·3430	[32]
**Outpatient unlinking**			
Spontaneous "unlinkage" from outpatient addiction care with MOUD	0·0481	0·0298-0·0666	[30,34]
Spontaneous "unlinkage" from outpatient addiction without MOUD	0·1560	0·1262-0·1860	[30,35]
**Mortality** *(weekly)*			
Background overdose subtracted mortality	Varies by age and sex	0·0008-0·0011	[36,37]
Probability of death, untreated IE	0·1623	0·0848-0·5358	[38,39]
Probability of death, untreated SSTI	0·0023	0·0023-0·0028	[39]
Probability of death, inpatient with IE	0·0100	0·0018-0·0161	[39-43]
Probability of death, inpatient with SSTI	0·0008	0·0008-0·0025	[39]
Probability of death, inpatient with overdose	0·0190	0·0130-0·0270	[44]
**Costs**± (weekly)			
Background costs	Varies by age and sex		[45]
No current injection drug use	$224	$112-$336	[28]
High frequency injection drug use	$357	$178-$536	[28]
Low frequency injection drug use	$238	$119-$357	[28]
Fatal overdose	$430	$215-$645	[46]
Non-fatal overdose not hospitalized	$1,118	$559-$1678	[46]
Hospitalization for IE	$21,573	$8,736-$34,410	[16]
Hospitalization for SSTI	$17,751	$9,124-$26,378	[16]
Hospitalization for overdose	$14,195	$12,744-$15,646	[46]
ACS	$225	$150-$300	Unpublished BMC ACS data, [47]
Outpatient addiction visit with MOUD	$81	$78-$138	[47,48]
Outpatient addiction visit without MOUD	$81	$62-$138	[28,47,48]

Notes: The ReDUCE Model runs on a weekly time cycle, therefore, all probabilities in this table are weekly probabilities.

IE=infective endocarditis; SSTI=skin and soft tissue infections; AMA=against medical advice; MOUD=medication for opioid use disorder; ACS=addiction consult service

*Expert communication with Drs. Honora Englander and Caroline King

**ALIVE = AIDS Linked to the IntraVenous Experience

*** These transition probabilities were derived from a combination of unpublished data from Oregon Health Sciences University, Boston Medical Center, and the Veteran's Administration. Further explanations are provided in the Supplemental Appendix.

**** Estimates were derived from 2015 to 2016 NYC provisional overdose data

±In the U.S., costs of medical care may fall on the individual, payor, or individual hospital. We performed this analysis from the perspective of the general payor system. Medical costs include hospitalizations, outpatient care such as primary care and specialists, medications, and ambulance services (when applicable)

For the base case, we initiated the model such at all individuals were using "unsafe" injection practices. For the alternate scenario analysis, we assumed that no individuals were reusing needles and that all individuals were cleaning their skin regularly. We made the conservative assumption that "harm reduction services" had no impact on overdose, only on injection practices.

Supplemental Table 2. Calibration targets

Target	Target Value	Modeled Value	Standard Error
**1-year endocarditis among PWID**	55 per 10,000	55 per 10,000	5·80%
**1-year fatal overdose among PWID**	68 per 10,000	70 per 10,000	0·74%
**3-year IE mortality among PWID**	29·0%	35·0%	17·4%
**Remaining life expectancy among PWID**	35·3 years	35·5 years	0·62%

PWID = People who inject drugs

Supplemental Table 3. Weekly probability of infection (endocarditis and skin and soft tissue), stratified by injection behavior profile

Injection behavior profile	Value (mean, standard deviation)
**High frequency, higher infection risk**	0·0045, 0·067
**High frequency, lower infection risk**	0·0004, 0·020
**Low frequency, higher infection risk**	0·0023, 0·048
**Low frequency, lower infection risk**	0·0002, 0·014

Note: higher infection risk=persons who do not clean their skin and reuse injection equipment; lower infection risk=persons who clean their skin and do not reuse injection equipment

Supplemental Table 4. Impact inventory

Sector	Type of Impact	Perspective	Notes
		Third-Party Payer	
**Formal Health Care**			
Health	**Health Outcomes (Effects)**		
	Longevity	√	
	HRQoL	√	
	Other Health Effects	√	
	**Medical Costs**		
	Third-Party Payers	√	
	Patients out-of-pocket		
	Future related medical costs	√	
	Future unrelated medical costs		
**Informal Health Care**			
Health	Patient-time costs	N/A	
	Unpaid caregiver-time costs	N/A	
	Transportation costs	N/A	
**Non-Health Care Sectors**			
Productivity	Labor market earnings lost	N/A	
	Cost of unpaid lost productivity	N/A	
	Cost of uncompensated household production	N/A	
Consumption	Future consumption unrelated to health	N/A	
Social Services	Cost of social services related to intervention	N/A	
Legal or criminal justice	Number of crimes related to intervention	N/A	
	Cost of crimes related to intervention	N/A	
Education	Impact on educational achievement	N/A	
Housing	Cost of intervention on home improvements	N/A	
Environment	Production of toxic waste by intervention	N/A	
Other	Other impacts	N/A	

HRQoL: Health-related quality of life; Impact Inventory included on recommendation from the Second Panel on Cost-Effectiveness in Health and Medicine

Supplemental Table 5. CHEERS checklist

Element	Journal Article	Supplement
**Introduction**		
Background of the problem	√	
**Study Design and Scope**		
Objectives	√	
Audience	√	
Type of analysis	√	
Target population	√	
Description of intervention and comparators	√	
Other intervention descriptors	√	
Boundaries of the analysis; defining the scope or comprehensiveness of the study	√	
Time horizon	√	
Analytic perspectives	√	
Whether this analysis meets the requirements of the reference case	√	
Analysis plan	√	
**Methods and data**		
If model based analysis:		
Description of event pathway or model	√	
Model diagram	√	
Description of the model used	√	
Modeling assumptions		√
Software used		√
Identification of key outcomes	√	
Complete information on sources of effectiveness data, cost data, and preference weights	√	
Methods for obtaining estimates of effectiveness (including approaches used for evidence synthesis)		√
Methods for obtaining estimates of costs and preference weights		√
Critique of data quality		√
Statement of costing year	√	
Statement of methods used to adjust for inflation	√	
Statement of currency used	√	
Source of expert judgment (if applicable)	√	
Statement of discount rates	√	
**Impact Inventory**		
Full accounting of consequences within and outside the health care sector	√	
**Results**		
Results of model validation		√
Reference case results: total costs and effectiveness, incremental costs and effectiveness, ICERs, uncertainty measures	√	
Disaggregated results for important categories of costs, outcomes or both	√	
Results of sensitivity analysis	√	√
Other estimates of uncertainty	√	√
Graphical representation of cost-effectiveness results	√	
Graphical representation of uncertainty analyses	√	√
Aggregate cost and effectiveness information	√	
Secondary analyses	√	√
**Disclosures**		
Statement of any potential conflicts of interest due to funding source, collaborations, or outside interests	√	
**Discussion**		
Summary of reference case results	√	
Discussion of the study results in the context of results of related cost-effectiveness analyses	√	

Discussion of ethical implications	√	
Limitations of the study	√	
Relevance of study results to specific policy questions or decisions	√	
Summary of sensitivity of results to assumptions and uncertainties in the analysis	√	

Reporting checklist included on recommendation from the Second Panel on Cost-Effectiveness in Health and Medicine

Supplemental Table 6. Consolidated Health Economic Evaluation Reporting Standards (CHEERS) Checklist

Item	Item	Recommendation	Section
Title and Abstract			
Title	1	Identify the study as an economic evaluation	Title
Abstract	2	Provide a structured summary of objectives, perspective, setting, methods, results, and conclusions	Abstract
Introduction			
Background and objectives	3	Provide an explicit statement of the broader context for the study	Introduction
Methods			
Target population and subgroups	4	Describe characteristics of the base case population and subgroups analyzed, including why they were chosen	Introduction
Setting and location	5	State relevant aspects of the system in which decisions need to be made	Methods: Overview
Study perspective	6	Describe the perspective of the study and relate this to the costs being evaluated	Methods: Overview
Comparators	7	Describe the interventions or strategies being compared and state why they were chosen	Methods: Overview
Time horizon	8	State the time horizons over which costs and consequences are being evaluated	Methods: Overview
Discount rate	9	Report/explain the choice of discount rate used for costs and outcomes	Methods: Overview
Choice of health outcomes	10	Describe what outcomes were used as the measure of benefit in the evaluation and their relevance for the analysis	Methods: Overview
Measurement of effectiveness	11	Describe fully the methods used for identification of included studies and synthesis of clinical effectiveness data	Methods: Overview
Measurement and valuation of preference based outcomes	12	If applicable, describe the population and methods used to elicit preferences for outcomes	Methods: Overview
Estimating resources and costs	13	Describe approaches and data sources used to estimate resource use associated with model health states	Supplement
Currency, price date and conversion	14	Report the dates of the estimated resource quantities and unit costs	Methods: Overview
Choice of model	15	Describe and give reasons for the specific type of decision-analytical model used	Methods: Model Description; Supplement
Assumptions	16	Describe all structural or other assumptions underpinning the decision-analytical model	Methods: Model Description; Supplement
Analytical methods	17	Describe all analytical methods supporting the evaluation	Methods: Model Description; Supplement
Results			
Study parameters	18	Report the values, ranges, references, and probability distributions for all parameters	Table 1
Incremental costs and outcomes	19	For each intervention, report mean values for the main categories of estimated costs and outcomes of interest, as well as mean differences between comparator groups	Tables 2
Characterizing uncertainty	20	Describe the effects on the results of uncertainty for all input parameters and uncertainty related to the structure of the model and assumptions	Results: Sensitivity Analysis; Supplement
Characterizing heterogeneity	21	If applicable, report differences in costs, outcomes, or cost-effectiveness that can be explained by variations between subgroups of patients	Results: Sensitivity Analysis; Supplement

Discussion			
Findings, limitations, generalizability, and current knowledge	22	Summarize key findings and describe how they support the conclusions reached, and limitations to generalizability	Discussion
Other			
Source of funding	23	Describe study funding and other non-monetary sources of support	Methods: Role of the Funding Source
Conflicts of interest	24	Describe any potential conflicts of interest	Author Funding/Conflict Statements

Supplemental Table 7. Selected cost and clinical outcomes for alternate scenario analysis under ideal harm reduction provision

Strategy[†]	Average per person cost *$*	Life Expectancy *LY*	Hospitalizations averted/10,000[‡]	Fatal overdoses averted/ 10,000
**Status quo**	$754,400 ($565,224-$920,475)	74·98 (72-76)	--	--
**MOUD with bridge**	$754,600 ($565,236-$921,064)	74·99 (72-76)	13 (6-14)	5 (3-8)
**ACS alone**	$755,000 ($566,430-$922,144)	75·01 (72-76)	19 (-1-27)	8 (2-16)
**MOUD + ACS**	$755,200 ($566,024-$921,837)	75·02 (72-76)	21 (0-32)	9 (3-18)

[*]All values in this table are undiscounted

Supplemental Table 8. Results of cost effectiveness analysis for alternate scenario

Strategy†	Average Discounted Cost *$* (95% Credible Interval)	Incremental Average Discounted Cost *$*	Discounted Life Years (95% Credible Interval)	Incremental Discounted Life Expectancy	ICER* ($/LY)
**Status quo**	$422,080 ($318,172-$520,219)	--	19·27 (17·87-19·83)	--	--
**MOUD with bridge**	$422,130 ($318,114-$520,397)	$50	19·28 (17·89-19·84)	0·01	$10,100
**ACS alone**	$422,290 ($318,745-$520,919)	$160	19·29 (17·90-19·84)	0·01	Dominated
**MOUD + ACS**	$422,320 ($318,451-$520,760)	$30	19·30 (17·90-19·84)	0·01	$20,100

*The overall incremental cost-effectiveness ratio was calculated as the difference in the average discounted costs for the total U.S. population divided the difference in the discounted quality adjusted life expectancy for the total U.S. population, all discounted at 3% per year.

LY = life years; ICER = incremental cost effectiveness ratio

Supplemental Table 9. Clinical outcomes in the Base Case Scenario among a cohort of 5 million people over 100 years

	No inpatient interventions	MOUD Only	ACS Only	MOUD and ACS
total cases of fatal overdose	498753	480658	469679	466194
total cases of non-fatal overdose	3325608	3209624	3139977	3116445
total cases of skin and soft tissue infections	11223125	10828562	10609085	10540479
total cases of infective endocarditis	2735765	2609685	2524124	2505210
total cases of cured skin and soft tissue infections	10345476	10002299	9821089	9759469
total cases of cured infective endocarditis	1658477	1598029	1577099	1567657
total number of people who got hospitalized	3376877	3377335	3377827	3377694
total cases of hospitalizations	12634310	12230228	12041049	11970934
total cases of hospitalizations for overdose	323064	312525	305201	303019
total cases of hospitalizations for skin and soft tissue infections	10924451	10561679	10370417	10306318
total cases of hospitalizations for infective endocarditis	2137119	2059694	2032169	2020415
IDU Mortality attributable to skin and soft tissue infections	362206	342816	329422	327285
IDU Mortality attributable to infective endocarditis	1062853	997999	933851	924596

Supplemental Figure 1. Total discounted costs and life years for status quo, expanded MOUD, ACS, and combined treatment strategies from payer perspective

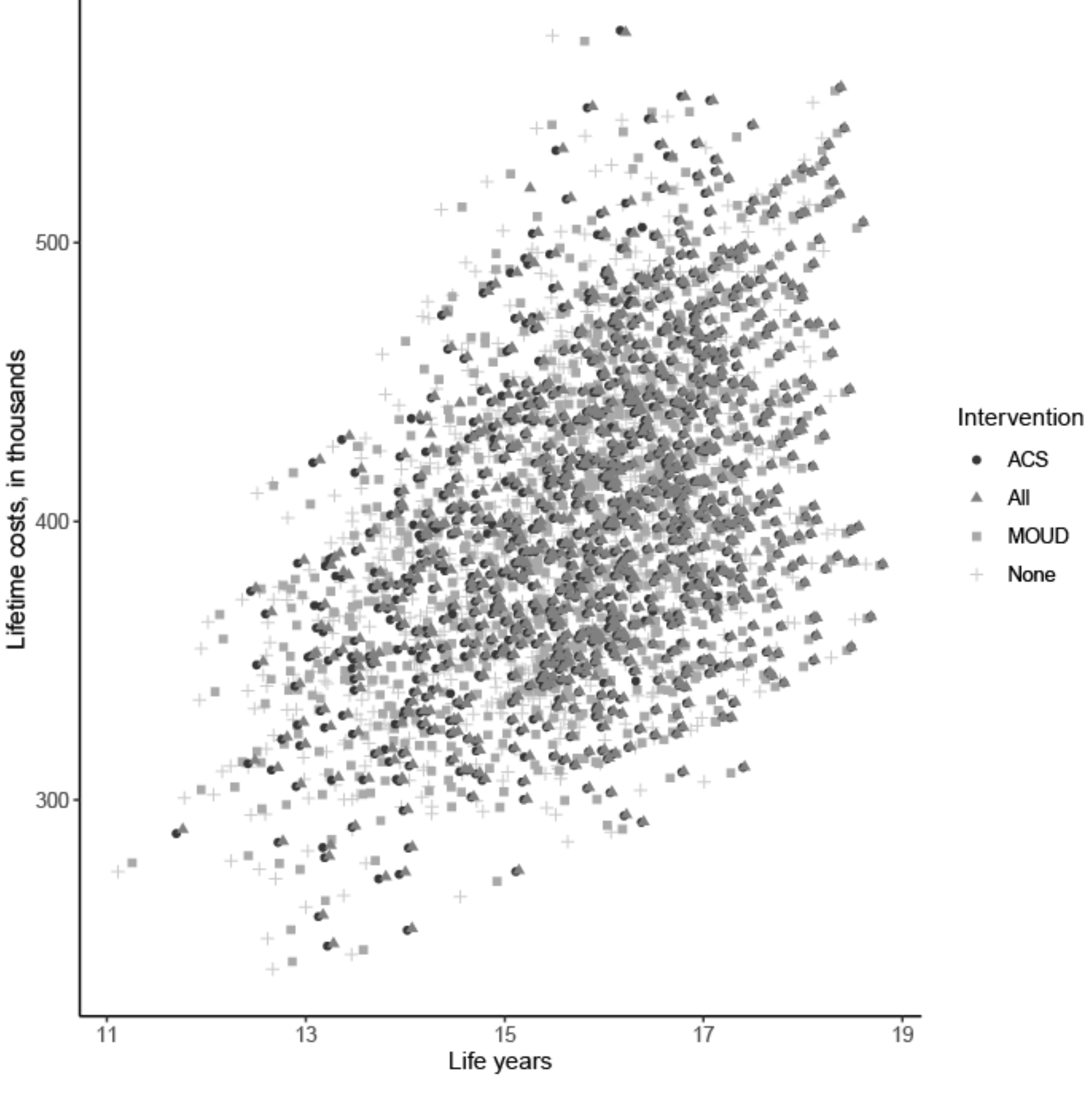

Plotted from individual results of 990 model runs in probabilistic sensitivity analysis. Status quo: standard hospital care-detoxification for opioids, no addiction consult service (grey cross); MOUD with bridge: expanded inpatient MOUD prescribing (grey square); ACS alone: implementation of addiction consult services (black circle); Combined: combined MOUD with ACS strategy (grey triangle).

Supplemental Figure 2. Cost-effectiveness plane for MOUD only strategy vs. status quo from payer perspective

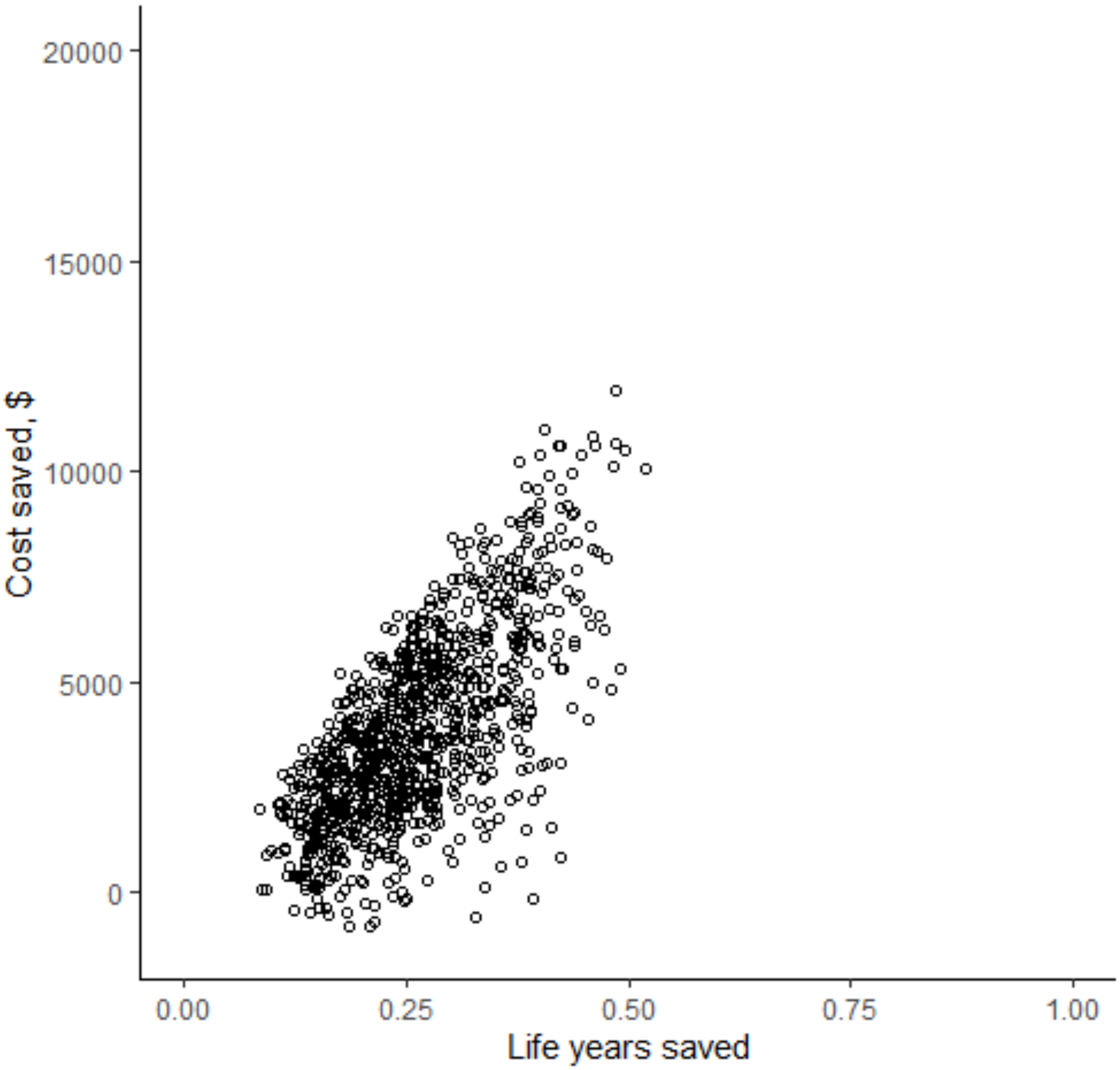

Plotted from individual results of 1000 model runs in probabilistic sensitivity analysis. Status quo: standard hospital care-detoxification for opioids, no addiction consult service; MOUD with bridge: expanded inpatient MOUD prescribing.

Supplemental Figure 3. Cost-effectiveness plane for ACS only strategy vs. status quo from payer perspective

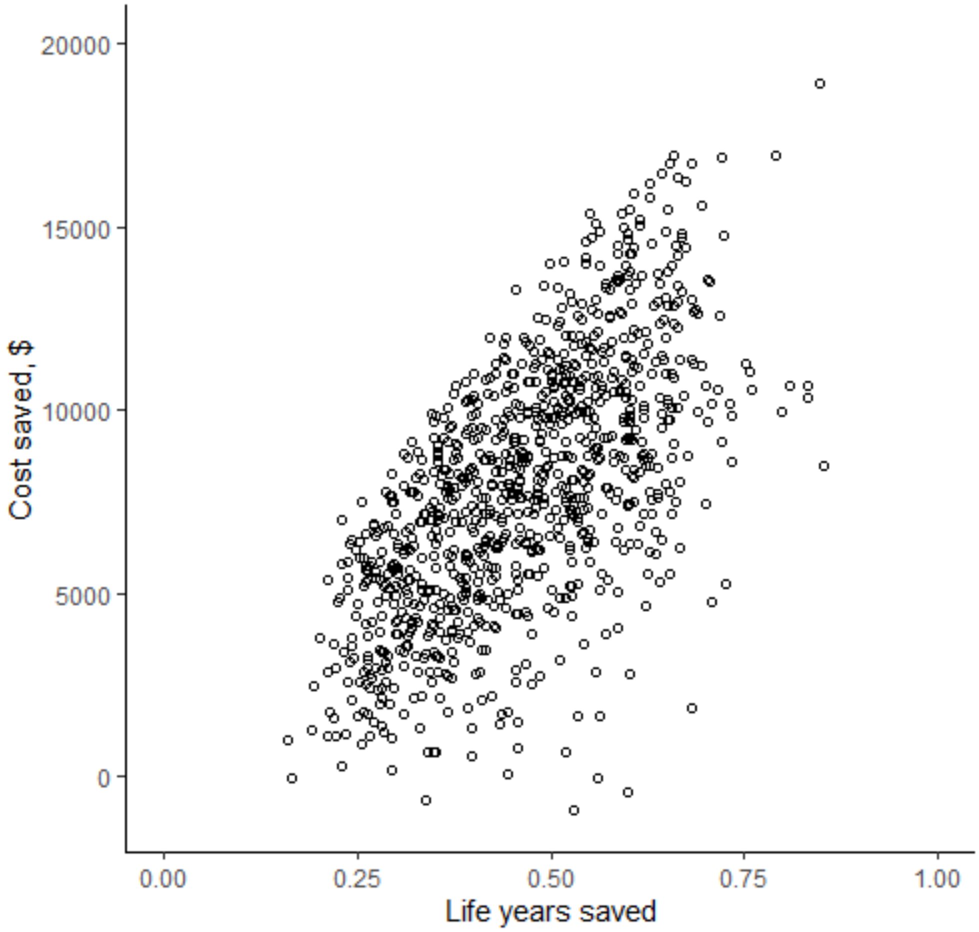

Plotted from individual results of 1000 model runs in probabilistic sensitivity analysis. Status quo: standard hospital care-detoxification for opioids, no addiction consult service; ACS alone: implementation of addiction consult services.

Supplemental Figure 4. Cost-effectiveness plane for combined ACS and MOUD strategy vs. status quo from payer perspective

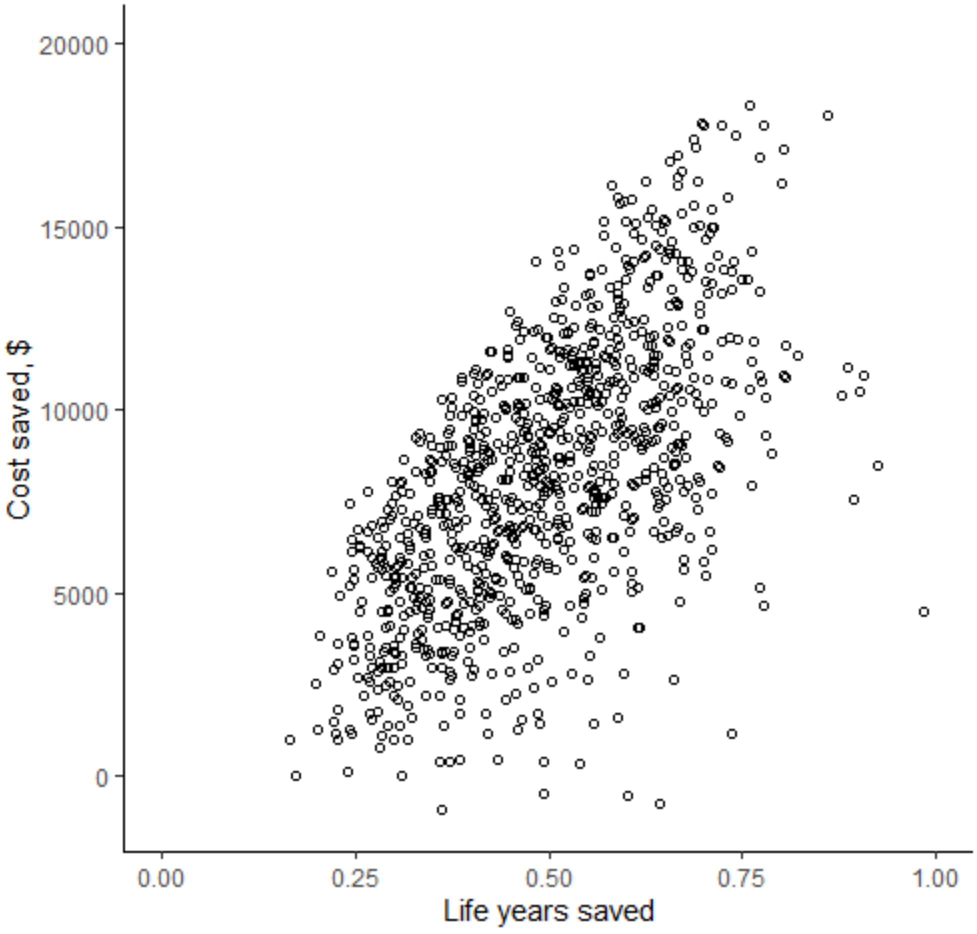

Plotted from individual results of 1000 model runs in probabilistic sensitivity analysis. Status quo: standard hospital care-detoxification for opioids, no addiction consult service; combined MOUD with ACS strategy.

Supplemental Figure 5. Total discounted costs and life years for status quo, expanded MOUD, ACS, and combined treatment strategies from payer perspective for alternative scenario

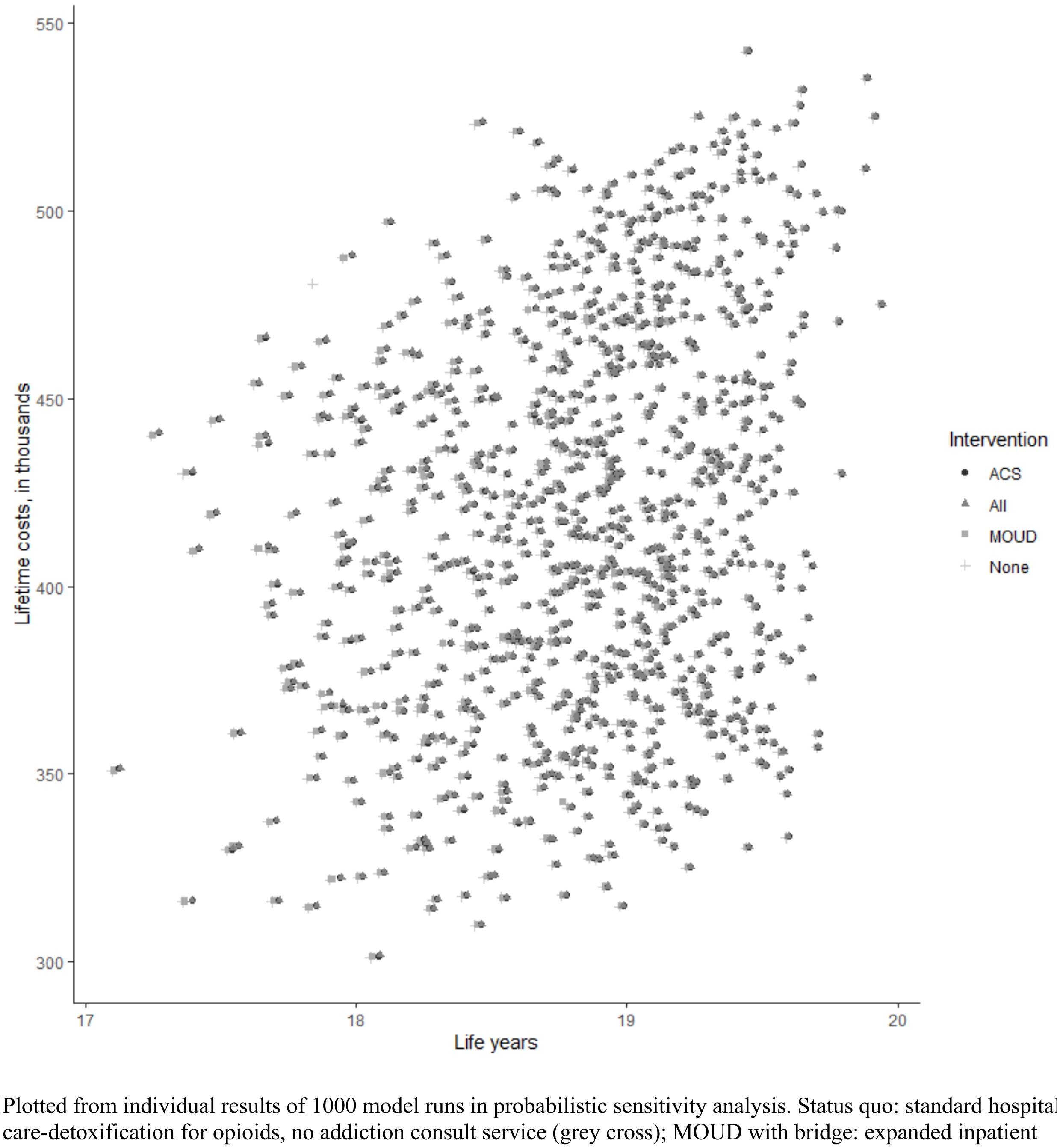

Plotted from individual results of 1000 model runs in probabilistic sensitivity analysis. Status quo: standard hospital care-detoxification for opioids, no addiction consult service (grey cross); MOUD with bridge: expanded inpatient MOUD prescribing (grey square); ACS alone: implementation of addiction consult services (black circle); Combined: combined MOUD with ACS strategy (grey triangle).

Supplemental Figure 6. Cost-effectiveness plane for MOUD only strategy vs. status quo from payer perspective for the alternate scenario analysis

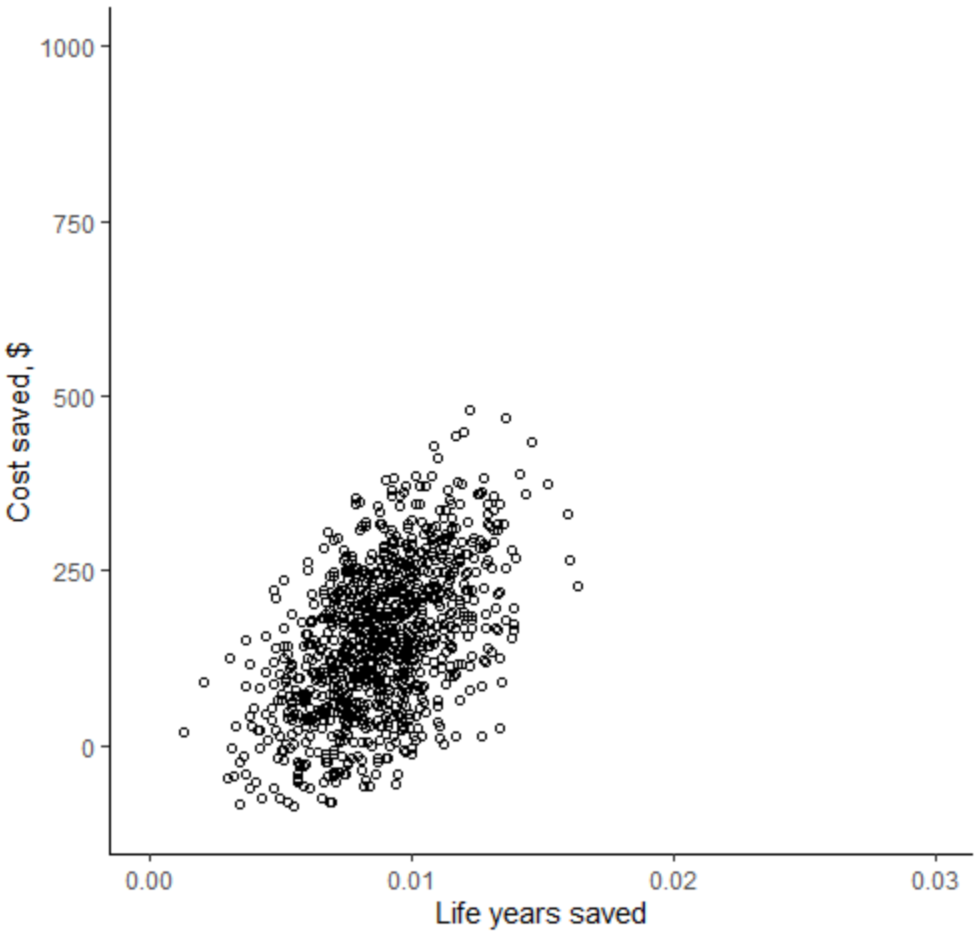

Plotted from individual results of 1000 model runs in probabilistic sensitivity analysis. Status quo: standard hospital care-detoxification for opioids, no addiction consult service; MOUD with bridge: expanded inpatient MOUD prescribing.

Supplemental Figure 7. Cost-effectiveness plane for ACS only strategy vs. status quo from payer perspective for the alternate scenario analysis

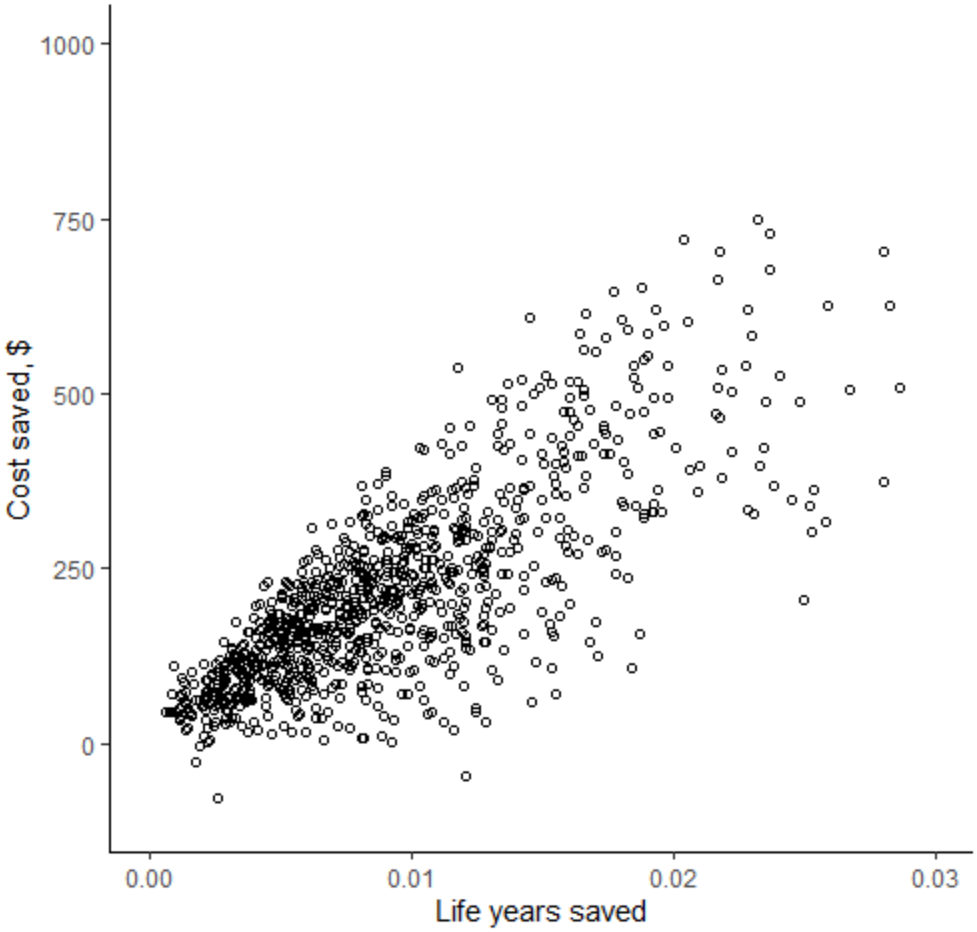

Plotted from individual results of 1000 model runs in probabilistic sensitivity analysis. Status quo: standard hospital care-detoxification for opioids, no addiction consult service; ACS alone: implementation of addiction consult services.

Supplemental Figure 8. Cost-effectiveness plane for combined ACS and MOUD strategy vs. status quo from payer perspective for the alternate scenario analysis

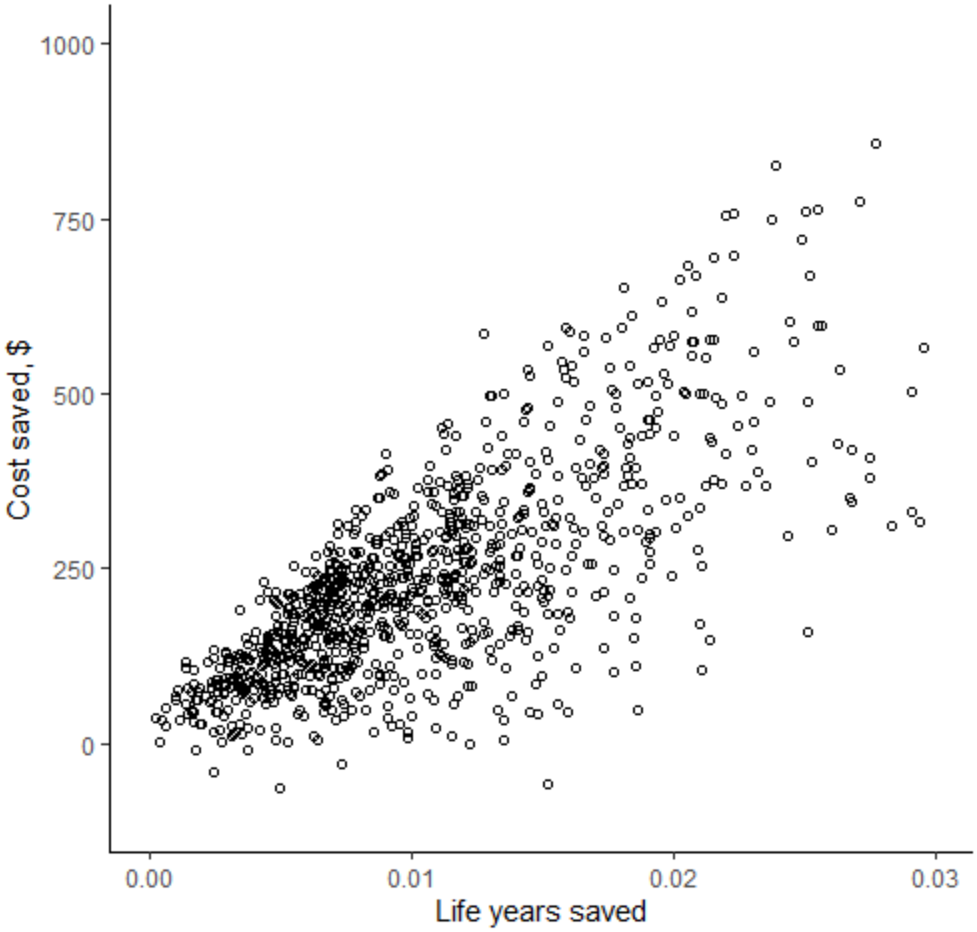

Plotted from individual results of 1000 model runs in probabilistic sensitivity analysis. Status quo: standard hospital care-detoxification for opioids, no addiction consult service; MOUD with bridge: expanded inpatient MOUD prescribing.

Supplemental Figure 9. Cost-effectiveness acceptability curves for modeled treatment strategies for the alternate scenario analysis

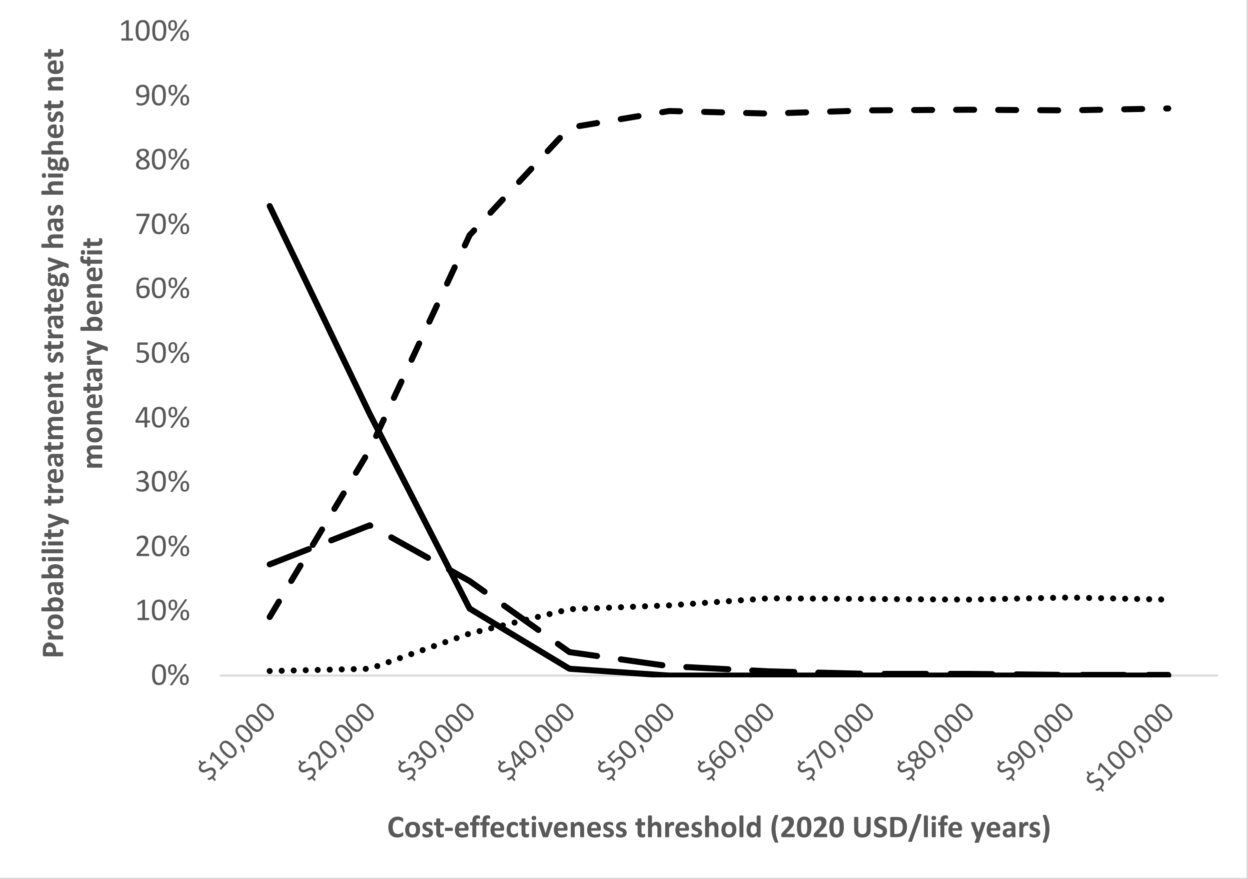

Status quo: solid black line; MOUD with bridge: large dashed black line; ACS alone: dotted black line; Combined: small dashed black line

Supplemental Figure 10. Flow diagram of the hospitalization module with probabilities

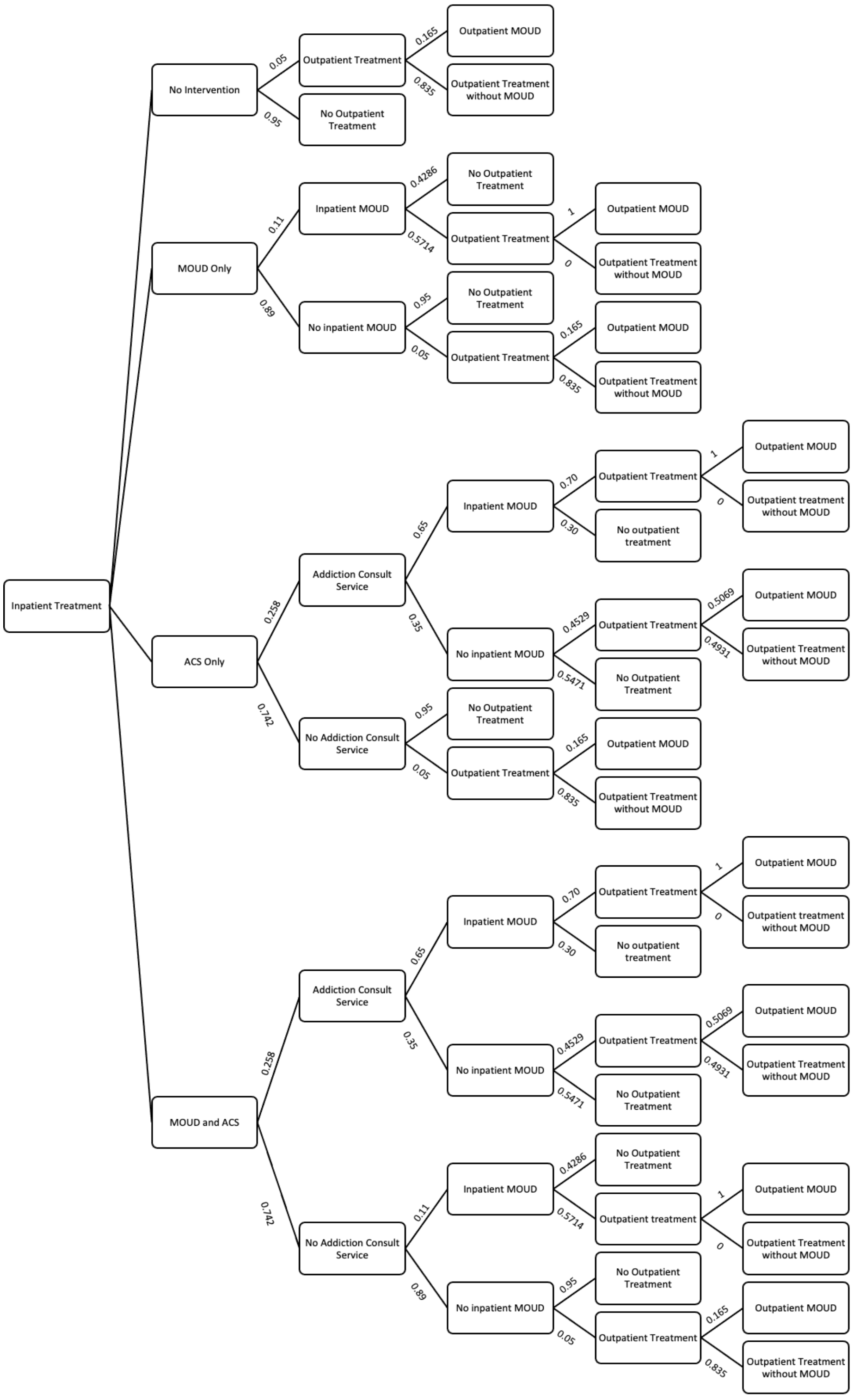

References

1. Lansky A, Finlayson T, Johnson C, et al. Estimating the number of persons who inject drugs in the united states by meta-analysis to calculate national rates of HIV and hepatitis C virus infections. *PLoS One* 2014; **9**(5): e97596.
2. Martins SS, Sarvet A, Santaella-Tenorio J, Saha T, Grant BF, Hasin DS. Changes in US Lifetime Heroin Use and Heroin Use Disorder: Prevalence From the 2001-2002 to 2012-2013 National Epidemiologic Survey on Alcohol and Related Conditions. *JAMA Psychiatry* 2017; **74**(5): 445-55.
3. Degenhardt L, Peacock A, Colledge S, et al. Global prevalence of injecting drug use and sociodemographic characteristics and prevalence of HIV, HBV, and HCV in people who inject drugs: a multistage systematic review. *Lancet Glob Health* 2017; **5**(12): e1192-e207.
4. US Centers for Disease Control and Prevention. HIV Infection, Risk, Prevention, and Testing Behaviors among Persons Who Inject Drugs—National HIV Behavioral Surveillance: Injection Drug Use, 20 U.S. Cities, 2015. HIV Surveillance Special Report 18. Revised edition. http://www.cdc.gov/hiv/library/reports/hiv-surveillance.html. Published May 2018. Accessed January 2020.
5. US Census Bureau. Table DP05 - ACS Demographic and Housing Estimates. 2016.
6. Tan S, Makela S, Heller D, et al. A Bayesian evidence synthesis approach to estimate disease prevalence in hard-to-reach populations: hepatitis C in New York City. *Epidemics* 2018; **23**: 96-109.
7. Buresh M, Genberg BL, Astemborski J, Kirk GD, Mehta SH. Recent fentanyl use among people who inject drugs: Results from a rapid assessment in Baltimore, Maryland. *Int J Drug Policy* 2019; **74**: 41-6.
8. Hser YI, Mooney LJ, Saxon AJ, et al. High Mortality Among Patients With Opioid Use Disorder in a Large Healthcare System. *J Addict Med* 2017; **11**(4): 315-9.
9. Hudgins R, McCusker J, Stoddard A. Cocaine use and risky injection and sexual behaviors. *Drug Alcohol Depend* 1995; **37**(1): 7-14.
10. Cedarbaum ER, Banta-Green CJ. Health behaviors of young adult heroin injectors in the Seattle area. *Drug Alcohol Depend* 2016; **158**: 102-9.
11. Massachusetts Department of Public Health. Opioid-related EMS Transports Massachusetts Residents: 2013–2015. Boston, MA: Commonwealth of Massachusetts, Department of Public Health; 5, 2016.
12. Massachusetts Department of Public Health. Data Brief: Opioid-Related Overdose Deaths among Maccachusetts Residents. Boston, MA: Commonwealth of Massachusetts, Department of Public Health; 11, 2019.
13. Hedegaard H, Miniño AM, Warner M. Drug overdose deaths in the United States, 1999–2017. NCHS Data Brief, no 329. Hyattsville, MD: National Center for Health Statistics. 2018.
14. An Assessment of Fatal and Nonfatal Opioid Overdoses in Massachusetts (2011-2015): Massachusetts Department of Public Health, 2017.
15. Larney S, Peacock A, Mathers BM, Hickman M, Degenhardt L. A systematic review of injecting-related injury and disease among people who inject drugs. *Drug Alcohol Depend* 2017; **171**: 39-49.
16. Miller AC, Polgreen PM. Many Opportunities to Record, Diagnose, or Treat Injection Drug-related Infections Are Missed: A Population-based Cohort Study of Inpatient and Emergency Department Settings. *Clin Infect Dis* 2019; **68**(7): 1166-75.
17. Hope V, Kimber J, Vickerman P, Hickman M, Ncube F. Frequency, factors and costs associated with injection site infections: findings from a national multi-site survey of injecting drug users in England. *BMC Infect Dis* 2008; **8**: 120.
18. Binswanger IA, Kral AH, Bluthenthal RN, Rybold DJ, Edlin BR. High prevalence of abscesses and cellulitis among community-recruited injection drug users in San Francisco. *Clin Infect Dis* 2000; **30**(3): 579-81.
19. Phillips KT, Stein MD. Risk practices associated with bacterial infections among injection drug users in Denver, Colorado. *Am J Drug Alcohol Abuse* 2010; **36**(2): 92-7.
20. Hope VD, Hickman M, Parry JV, Ncube F. Factors associated with recent symptoms of an injection site infection or injury among people who inject drugs in three English cities. *Int J Drug Policy* 2014; **25**(2): 303-7.
21. Jackson KA, Bohm MK, Brooks JT, et al. Invasive Methicillin-Resistant Staphylococcus aureus Infections Among Persons Who Inject Drugs - Six Sites, 2005-2016. *MMWR Morb Mortal Wkly Rep* 2018; **67**(22): 625-8.
22. Kadri AN, Wilner B, Hernandez AV, et al. Geographic Trends, Patient Characteristics, and Outcomes of Infective Endocarditis Associated With Drug Abuse in the United States From 2002 to 2016. *J Am Heart Assoc* 2019; **8**(19): e012969.

23. N'Guyen Y, Duval X, Revest M, et al. Time interval between infective endocarditis first symptoms and diagnosis: relationship to infective endocarditis characteristics, microorganisms and prognosis. *Ann Med* 2017; **49**(2): 117-25.
24. Hope VD, Ncube F, Parry JV, Hickman M. Healthcare seeking and hospital admissions by people who inject drugs in response to symptoms of injection site infections or injuries in three urban areas of England. *Epidemiol Infect* 2015; **143**(1): 120-31.
25. Caudarella A, Dong H, Milloy MJ, Kerr T, Wood E, Hayashi K. Non-fatal overdose as a risk factor for subsequent fatal overdose among people who inject drugs. *Drug Alcohol Depend* 2016; **162**: 51-5.
26. Alagna L, Park LP, Nicholson BP, et al. Repeat endocarditis: analysis of risk factors based on the International Collaboration on Endocarditis - Prospective Cohort Study. *Clin Microbiol Infect* 2014; **20**(6): 566-75.
27. Priest KC, Lovejoy TI, Englander H, Shull S, McCarty D. Opioid Agonist Therapy During Hospitalization Within the Veterans Health Administration: a Pragmatic Retrospective Cohort Analysis. *J Gen Intern Med* 2020; **35**(8):2365-2374.
28. Murphy SM, McCollister K.E.; Leff, J.A.; Yang, X.; Jeng, P.J.; Lee, J.D.; Nunes, E.V.; Novo, P.; Rotrosen, J.; Schackman, B.R. Cost-effectiveness of buprenorphine-naloxone versus extended-release naltrexone to prevent opioid relapse. *Annals of Internal Medicine* 2019; **170**(2): 90–98.
29. Englander H, King C, Nicolaidis C, et al. Predictors of Opioid and Alcohol Pharmacotherapy Initiation at Hospital Discharge Among Patients Seen by an Inpatient Addiction Consult Service. *J Addict Med* 2020; **14**(5): 415-22.
30. Liebschutz JM, Crooks D, Herman D, et al. Buprenorphine treatment for hospitalized, opioid-dependent patients: a randomized clinical trial. *JAMA Intern Med* 2014; **174**(8): 1369-76.
31. Trowbridge P, Weinstein ZM, Kerensky T, et al. Addiction consultation services - Linking hospitalized patients to outpatient addiction treatment. *J Subst Abuse Treat* 2017; **79**: 1-5.
32. Knudsen HK, Abraham AJ, Roman PM. Adoption and implementation of medications in addiction treatment programs. *J Addict Med* 2011; **5**(1): 21-7.
33. Larochelle MR, Bernson D, Land T, et al. Medication for Opioid Use Disorder After Nonfatal Opioid Overdose and Association With Mortality: A Cohort Study. *Ann Intern Med* 2018; **169**(3): 137-45.
34. Morgan JR, Schackman BR, Leff JA, Linas BP, Walley AY. Injectable naltrexone, oral naltrexone, and buprenorphine utilization and discontinuation among individuals treated for opioid use disorder in a United States commercially insured population. *J Subst Abuse Treat* 2018; **85**: 90-6.
35. Wakeman SE, Metlay JP, Chang Y, Herman GE, Rigotti NA. Inpatient Addiction Consultation for Hospitalized Patients Increases Post-Discharge Abstinence and Reduces Addiction Severity. *J Gen Intern Med* 2017; **32**(8): 909-16.
36. Chang KC, Wang JD, Saxon A, Matthews AG, Woody G, Hser YI. Causes of death and expected years of life lost among treated opioid-dependent individuals in the United States and Taiwan. *Int J Drug Policy* 2017; **43**: 1-6.
37. Arias E. United States life tables, 2008. *Natl Vital Stat Rep* 2012; **61**(3): 1-63.
38. Verhagen DW, Vedder AC, Speelman P, van der Meer JT. Antimicrobial treatment of infective endocarditis caused by viridans streptococci highly susceptible to penicillin: historic overview and future considerations. *J Antimicrob Chemother* 2006; **57**(5): 819-24.
39. Veldhuizen S, Callaghan RC. Cause-specific mortality among people previously hospitalized with opioid-related conditions: a retrospective cohort study. *Ann Epidemiol* 2014; **24**(8): 620-4.
40. Rodger L, Glockler-Lauf SD, Shojaei E, et al. Clinical Characteristics and Factors Associated With Mortality in First-Episode Infective Endocarditis Among Persons Who Inject Drugs. *JAMA Netw Open* 2018; **1**(7): e185220.
41. Cresti A, Chiavarelli M, Scalese M, et al. Epidemiological and mortality trends in infective endocarditis, a 17-year population-based prospective study. *Cardiovasc Diagn Ther* 2017; **7**(1): 27-35.
42. Hill EE, Herijgers P, Claus P, Vanderschueren S, Herregods MC, Peetermans WE. Infective endocarditis: changing epidemiology and predictors of 6-month mortality: a prospective cohort study. *Eur Heart J* 2007; **28**(2): 196-203.
43. Ternhag A, Cederstrom A, Torner A, Westling K. A nationwide cohort study of mortality risk and long-term prognosis in infective endocarditis in Sweden. *PLoS One* 2013; **8**(7): e67519.
44. Jiang Y, McDonald JV, Koziol J, McCormick M, Viner-Brown S, Alexander-Scott N. Can Emergency Department, Hospital Discharge, and Death Data Be Used to Monitor Burden of Drug Overdose in Rhode Island? *J Public Health Manag Pract* 2017; **23**(5): 499-506.
45. Agency for Healthcare Research and Quality. Medical Expenditure Panel Survey. 2020.

46. Behrends CN, Paone D, Nolan ML, et al. Estimated impact of supervised injection facilities on overdose fatalities and healthcare costs in New York City. *J Subst Abuse Treat* 2019; **106**: 79-88.
47. Centers for Medicare and Medicaid Services. Physician Fee Schedule. 2020. https://www.cms.gov/apps/physician-fee-schedule/search/search-criteria.aspx (accessed December 20, 2020).
48. Centers for Medicare and Medicaid Services. Clinical Laboratory Fee Schedule. 2020. https://www.cms.gov/Medicare/Medicare-Fee-for-Service-Payment/ClinicalLabFeeSched (accessed December 20, 2020).
